# Atmospheric Oxidation of NH_3_, HNO_3_ and NH_3_···HNO_3_ by OH, NH_2_, and NO_3_ Radicals. The Effect of Water Vapor

**DOI:** 10.1021/acs.jpca.5c06826

**Published:** 2026-01-07

**Authors:** Josep M. Anglada, Ramon Crehuet

**Affiliations:** 73039 Institute for Advanced Chemistry of Catalonia (IQAC) − CSIC, c/Jordi Girona 18- 26, E-08034 Barcelona, Spain.

## Abstract

Atmospheric ammonia, in both particulate and gaseous forms, has major ecological, health, and economic impacts, making it essential to understand its chemical processes. The reactions of ammonia and ammonia complexed with nitric acid with hydroxyl radical and the oxidation of nitric acid by amidogen radical and ammonia by nitrate radical, both taking into account the effect of water vapor, have been investigated using quantum mechanical (QCISD and CCSD­(T)) calculations with the 6-311+G­(2df,2p), aug-cc-pVTZ, and aug-cc-pVQZ and extrapolation to the CBS basis sets. From a mechanistic point of view, the reaction of NH_3_ + OH follows a conventional hydrogen transfer mechanism, but for the rest of reactions considered, the proton coupled electron transfer mechanism plays a key role. For the reaction of ammonia with hydroxyl radical we have computed a rate constant of 1.24 × 10^–13^ cm^3^·molecule^–1^·s^–1^ at 298 K, and the effect of water vapor is negligible. The calculated rate constant for the HNO_3_···NH_3_ + OH reaction is 6.50 × 10^–16^ cm^3^·molecule^–1^·s^–1^ at 298 K. and our results show that both HNO_3_ and NH_3_ moieties can be oxidized. The effect of water vapor on the oxidation of nitric acid by an amidogen radical is significant. We have computed a rate constant of 1.98 × 10^–13^ cm^3^·molecule^–1^·s^–1^, at 298 K and 100% of RH for the whole HNO_3_ + NH_2_ + H_2_O reaction, which is an 11% greater than the calculated value for the naked reaction. For the oxidation of ammonia by a nitrate radical, the effect of water vapor is huge. The calculated rate constant at 298 K and 100% of RH is 16 × 10^–15^ cm^3^·molecule^–1^·s^–1^ for the whole NH_3_ + NO_3_ + H_2_O reaction, that is, 751% greater than the value of the naked reaction at 298 K.

## Graphical abstract

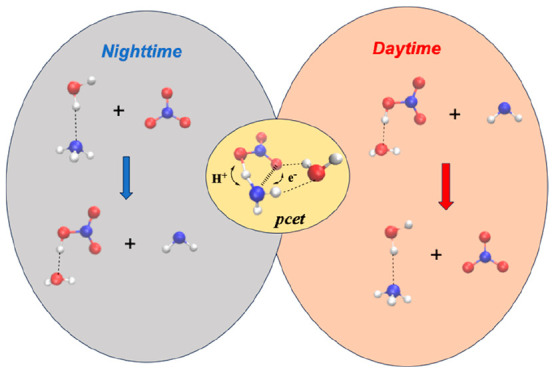

## Introduction

Ammonia is a major trace gas in the atmosphere. It is released from anthropogenic and biogenic sources, including agriculture, the use of fertilizers, the urine and manure produced by livestock, vehicle emissions, from wildfires and also from farming activities, and constitute the third most abundant nitrogen species in the troposphere.^1–5^ NH_3_ is the only alkaline gas in the atmosphere and plays an important role throughout the homogeneous and heterogeneous processes. Heterogeneously, ammonia acts as a precursor in the formation of tropospheric condensation nuclei, leading to aerosol formation.^6–8^ In particular, it reacts with atmospheric acid species such as nitric and sulfuric acids (originated from NO_x_ and SO_x_) producing fine particulate matter (PM_2.5_). From a homogeneous perspective, the reaction with hydroxyl radical (reaction 1) is the main factor responsible for the oxidation of ammonia, which leads to the formation of amidogen radical. This reaction is also important in the atmospheric formation and elimination of NO_x_ and in the combustion of fossil fuels.^9^

1 $${\mathrm{NH}}_{3}+\mathrm{OH}\to {\mathrm{NH}}_{2}+H_{2}O$$

Both particulate and gaseous ammonia have great ecological impact and important consequences in human and animal health^10–13^ with huge economic costs,^5^ and therefore it is of great interest to have a detailed knowledge of all possible chemical processes involving atmospheric NH_3_.

The amidogen radical product of reaction 1, can also oxidize nitric acid (reaction 2),^14,15^ which is an important inorganic acid in the Earth’s atmosphere. HNO_3_ is mainly produced by gas phase reaction of NO_2_ with OH in daytime and by hydrolysis of N_2_O_5_ in nighttime and it is removed by forming of aerosols, by contributing to the acid rain and by gas phase oxidation with OH radicals (equation 3).^16,17^

2 $${\mathrm{HNO}}_{3}+{\mathrm{NH}}_{2}\to {\mathrm{NO}}_{3}+{\mathrm{NH}}_{3}$$

3 $${\mathrm{HNO}}_{3}+\mathrm{OH}\to {\mathrm{NO}}_{3}+H_{2}O$$

Reactions 2 and 3 produce a nitrate radical, which is an important atmospheric oxidant in nighttime. We recently studied Reaction 2 ^14,15^ while reaction 3 is well known, both naked, and catalyzed by a water molecule.^18–22^ We proposed that reaction 2 competes with reaction 3,^14^ and due to the fact that reaction 2 regenerates ammonia, both reactions 1 and 2 form an atmospheric catalytic cycle.^14^

In a very recent work, Clark and co-workers^23^ suggest significant binding between ammonia and nitric acid, leading to long residence times for the NH_3_···HNO_3_ complexes, which could play a role in the first steps of condensation nucleation. In addition, field observations and laboratory investigations have revealed the existence of interdependence between atmospheric concentrations of nitric acid and ammonia,^24–28^ an therefore, one goal of this research is to study the reactivity of the NH_3_···HNO_3_ complex with hydroxyl radical (reaction 4) that can lead to the oxidation of the ammonia moiety, producing HNO_3_···H_2_O + NH_2_, or to the oxidation of nitric acid moiety, producing NH_3_···H_2_O + NO_3_.

4 $${\mathrm{NH}}_{3}\cdot \cdot \cdot \cdot {\mathrm{HNO}}_{3}+\mathrm{OH}\to \mathrm{products}$$

In addition, and provided the importance of water vapor in the troposphere, the second goal of this work is the study the effect of water on the previous reaction (reaction 2) and on the oxidation of ammonia by hydroxyl radical (reactions 1), resulting in reactions 5, 6, and 7 respectively.

5 $${\mathrm{HNO}}_{3}\cdot \cdot \cdot H_{2}O+{\mathrm{NH}}_{2.}\to \mathrm{products}$$

$${\mathrm{NH}}_{3}\cdot \cdot \cdot H_{2}O+{\mathrm{NO}}_{3}\to \mathrm{products}$$ 6

$$p{\mathrm{NH}}_{3}\cdot \cdot \cdot \cdot H_{2}O+\mathrm{OH}\to \mathrm{products}$$ 7

## Methods

The calculations carried out in this work have been performed using the quadratic configuration-interaction method with all single and double excitations (QCISD)^29^ and coupled-cluster calculations including all single and double excitations with a perturbative estimation of all connected triple excitations (CCSD­(T))^30–33^ . The basis sets employed in these calculations are the 6-311G­(d,p), 6-311+G­(2df,2p) basis set,^34,35^ and aug-cc-pVTZ and aug-cc-pVQZ basis sets.^36,37^

The reactions involving a water molecule and the reaction between HNO_3_···NH_3_ and OH show a great complexity in the area where the reactants interact forming pre-reactive structures, and in these cases, we have first explored the potential energy surface by employing the Nudged Elastic Band (NEB) approach,^38^ with the B3LYP functional^39^ and the 6-311+G­(2df,2p) basis set using the pDynamo library^40^ coupled to the Orca software.^41^ The stationary points found in this way were re-optimized at the QCISD level of theory with the same basis.

For the reactions between NH_3_ and OH and between NH_3_···H_2_O + OH, all stationary points have been obtained using the QCISD method and employing the 6-311+G­(2df,2p) basis set. At this level of theory, we have also performed harmonic vibrational frequency calculations to ascertain the nature (minima or saddle points) of the stationary points found on the PES, as well as to calculate the zero-point energy and the thermal contributions to the enthalpy and Gibbs free energy. In addition, we have verified the connectivity between a given transition state structure (TS) and the corresponding reactant and product by performing intrinsic reaction coordinate (IRC) calculations.^42–44^

For the reaction between HNO_3_···NH_3_ and OH we obtained all stationary points employing, in a first step, the QCISD approach with the 6-311G­(d,p) basis set. At this level of theory, we have characterized the nature of the corresponding stationary points by performing harmonic frequency calculations and we have carried out IRC calculations to verify the connectivity between the TS and the corresponding reactants and products. In a second step, all stationary points have been re-optimized using the QCISD methods with the 6-311+G­(2df,2p) basis set.

In order to obtain more reliable relative energies, we performed single point CCSD­(T) calculations using the geometries optimized at the QCISD/6-311+G­(2df,2p) level of theory. For the CCSD­(T) calculations we have employed the aug-cc-pVTZ and aug-cc-pVQZ basis sets and we have also considered the extrapolation to the complete basis set (CBS) according to scheme by Helgaker et al.^45^

To check the reliability of the single determinant based CCSD calculations, we have examined the value of the T1 diagnostic^46,47^ of the CCSD wave function. The T_1_ diagnostic gives a qualitative assessment of the significance of the possible multireference character of the wave function. The larger is the T_1_ diagnostic value, the less reliable are the results of the CCSD wave function, and values over 0.044 are considered no reliable.^47^ The values of the T1 diagnostic of the CCSD wave function of this work are below 0.035, indicating the accuracy of our results.

The quantum chemical calculations have been carried out by employing Gaussian-03,^48^ ORCA^41^ and *pDynamo programs*.^40^ The Molden^49,50^ and VMD^51^ programs have been used to visualize the geometric and electronic features of the different stationary points.

## Results and Discussion

### The NH_3_-OH, NH_3_-H_2_O, NH_3_-HNO_3_ and HNO_3_···H_2_O complexes

Figure 1 shows the most relevant geometrical parameters, and Table 1 contains their binding energies. Figure 1 shows that in the NH_3_···OH, NH_3_···H_2_O, and HNO_3_···NH_3_ complexes, the two moieties are held together by a single hydrogen bond which occurs between the lone pair over the nitrogen atom of ammonia moiety and one hydrogen atom of the other moiety, whereas in the HNO_3_···H_2_O the two moieties are held together by two hydrogen bonds, one between the lone pair of the water molecule and the hydrogen atom of the HNO_3_ moiety, and the other between one oxygen atom of nitric acid and one hydrogen atom of water.

**Figure 1:**
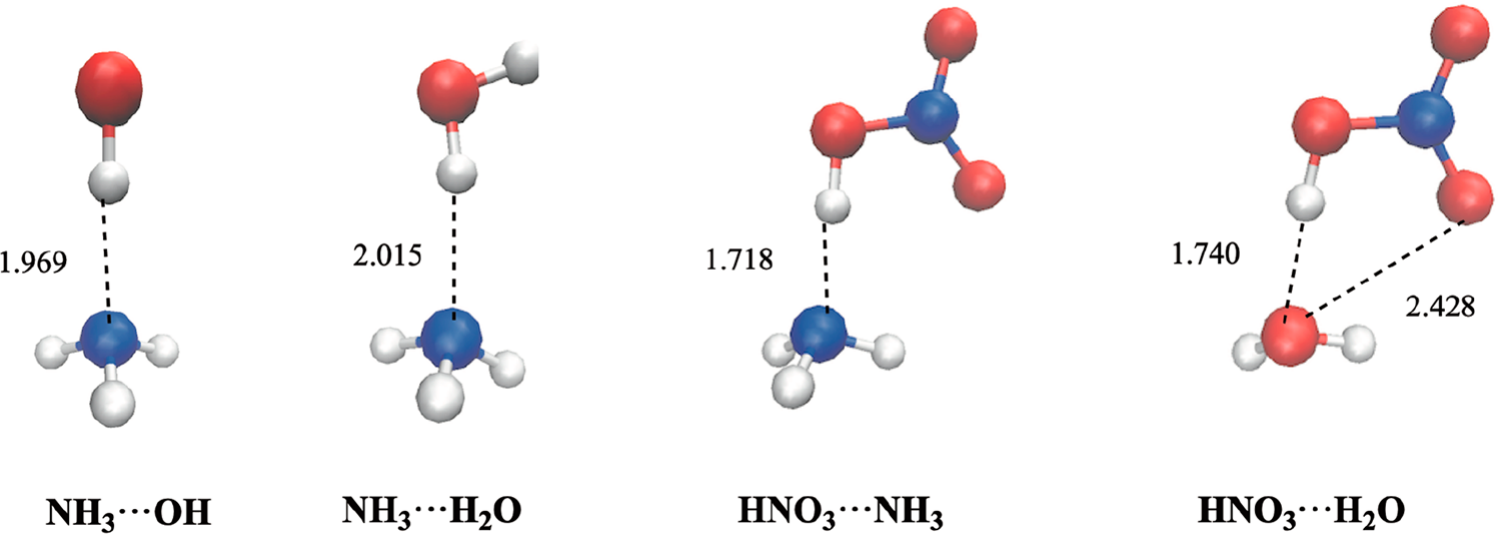
Relevant geometrical parameters of NH_3_···OH, NH_3_···H_2_O, HNO_3_···NH_3_, and HNO_3_···H_2_O complexes. Nitrogen atoms are in blue, oxygen atoms in read, and hydrogen atoms in white.

**Table 1:** Computed Binding Energies of Several Complexes of NH_3_ and HNO_3_ at 298 K and 1 atm^a^

	Δ*E*	Δ(*E*+ZPE)	Δ*H* (298 K)	Δ*G* (298 K)
$NH_{3}+OH\to NH_{3}\cdot \cdot \cdot OH\;(\mathrm{Ck})$	–7.74	–5.18	–6.05	1.26
$NH_{3}+H_{2}O\to NH_{3}\cdot \cdot \cdot H_{2}O$	–6.43	–4.18	–5.19	2.79
$NH_{3}+HNO_{3}\to NH_{3}\cdot \cdot \cdot HNO_{3}$	–13.43	–11.42	–11.73	–3.02
$HNO_{3}+H_{2}O\to HNO_{3}\cdot \cdot \cdot H_{3}O$	–10.25	–8.08	–8.40	–3.02

a Computed at CCSD­(T)/CBS//QCISD/6-311+G­(2df,2p). The ZPE, entropic and enthalpic corrections are computed at QCISD/6-311+G­(2df,2p).

It is worth reminding the reader that in any X-H···Y hydrogen bond interaction, X acts as a donor and Y as an acceptor so that the hydrogen bond complex can be seen as an incipient proton transfer from X to Y. Moreover, there is an associated electron transfer whose direction is reverse to the direction of the proton donation.^52^ This charge transfer is provided by the interaction of the lone pair of the accepting atom with the unoccupied σ* orbital of the X-H donor,^53^ and the most stable a complex is, the larger its charge transfer and the shorter its hydrogen bond length.

These complexes have already been reported in the literature (refs 22, 23, 27, 54–60 ), and therefore in this work we will only discuss the most relevant trends. Table 1 shows that the binding energies range between 4.18 kcal·mol^–1^ and 11.42 kcal·mol^–1^, and the H···N bond length ranges between 2.015 and 1.718 Å (see Figure 1). For the NH_3_···H_2_O, complex our calculations predict the largest OH···N hydrogen bond length (2.015 Å) and a N···O bond distance of 2.975 Å, in excellent agreement with experimental (2.989 Å)^56^ and calculated values from the literature (2.948 Å),^58^ and our computed binding energy is 4.18 kcal·mol^–1^. Interestingly the NH_3_···OH complex is 1 kcal·mol^–1^ more stable than the ammonia–water complex and the hydrogen bond length is shorter (1.969 Å), showing that the hydroxyl radical behaves as a slightly stronger acid than water. According to these features, the charge transfer associated to the hydrogen bond is smaller in the NH_3_···H_2_O complex (18.3 millielectron (me) from water to ammonia), than in the NH_3_···OH complex (16.1 me from the OH moiety to the NH3 moiety). The NH_3_···HNO_3_ is the most stable complex, with a quite large computed binding energy of 11.42 kcal·mol^–1^, and has a shorter hydrogen bond length (1.718 Å), in good agreement with the results reported by Clark et al., although these authors predict a slightly smaller binding energy (10.2 kcal·mol^–1^).^23^ According to the values, the charge transfer associated with the hydrogen bond interaction is much larger (8.8 me from the NH_3_ moiety to the HNO_3_ moiety), which is in agreement with the greater acid character of HNO_3_. For the HNO_3_···H_2_O we have computed a binding energy of 8.08 kcal·mol^–1^, and one short hydrogen bond (1.740 Å), and one long hydrogen bond (2.428 Å), in good agreement with the values reported in the literature.^22,60,61^ for this complex we have calculated a small charge transfer, of 2.5 me, occurring from the water moiety to the nitric acid moiety, which is due to the fact that the two hydrogen bonds have opposite interactions.

In Table S2 we collected the calculated equilibrium constants of these complexes, which were computed in the range of temperatures between 220 and 320 K. At 298.15 K, our computed values read 1.47 × 10^–20^, 2.20 × 10^–21^, 2.05 × 10^–17^, and 1.72 × 10^–19^ cm^3^·molecules^–1^, for NH_3_···OH, NH_3_···H_2_O, NH_3_···HNO_3_, and HNO_3_···H_2_O, respectively. For the ammonia–nitric acid complex our predicted values are slightly larger than have been reported previously^23^ (2.30 times at 300 K), which is due to the larger binding energy computed in our work.

The NH_3_···H_2_O and HNO_3_···NH_3_ and HNO_3_···H_2_O complexes have potential atmospheric interest, and our calculations allow estimation of their atmospheric abundance. Field observations have reported atmospheric concentration of nitric acid and ammonia ranging between 1.16 × 10^10^ and 1.32 × 10^12^ molecule·cm^–3^ for nitric acid,^62,63^ and in the range between 1.97 × 10^11^ and 3.74 × 10^12^ molecule·cm^–3^ for ammonia,^63–65^ while measures on mean simultaneous concentrations of ammonia and nitric acid, observed indicate that the ratio [NH_3_]/[HNO_3_] ranges between 17 and 64 times.^63,66–68^ Considering these values, the water concentrations ranging between 2.58 × 10^17^ and 2.35 × 10^18^ molecules·cm^–3^ at 100% RH and between 280 and 320 ^69^ and the equilibrium constants reported in Table S1, we can predict that, at very hot and humid conditions, the NH_3_···H_2_O, NH_3_···HNO_3_, and HNO_3_···H_2_O complexes can reach atmospheric concentrations up to 1.13 × 10^10^, 1.01 × 10^8^, and 1.78 × 10^11^ molceucles·cm^–3^, respectively.

### The Reaction between NH_3_ and OH and the Effect of Water Vapor

The gas phase reaction of ammonia was carried out by hydroxyl radical. This reaction is also a prototype of the hydrogen atom abstraction processes by radicals and has been investigated in the literature,^70–73^ along with the effect of water vapor on this reaction.^74^ In this work, we will briefly discuss the main trends of these reactions, and we refer the reader to these references for a more complete description of their features. In Figure 2, we have plotted a scheme of the potential energy surface of both reactions and in Table 2 we have collected the corresponding relative energies. We have named the stationary points of the naked reaction starting by the letter A, followed by the acronym CR for the pre-reactive complexes, TS for the transition states, and CP for the post reactive complex, followed by a number. For the reaction with one water molecule, the stationary points are named starting by letter B, followed by CR, TS, or CP and a number in the same manner.

**Figure 2:**
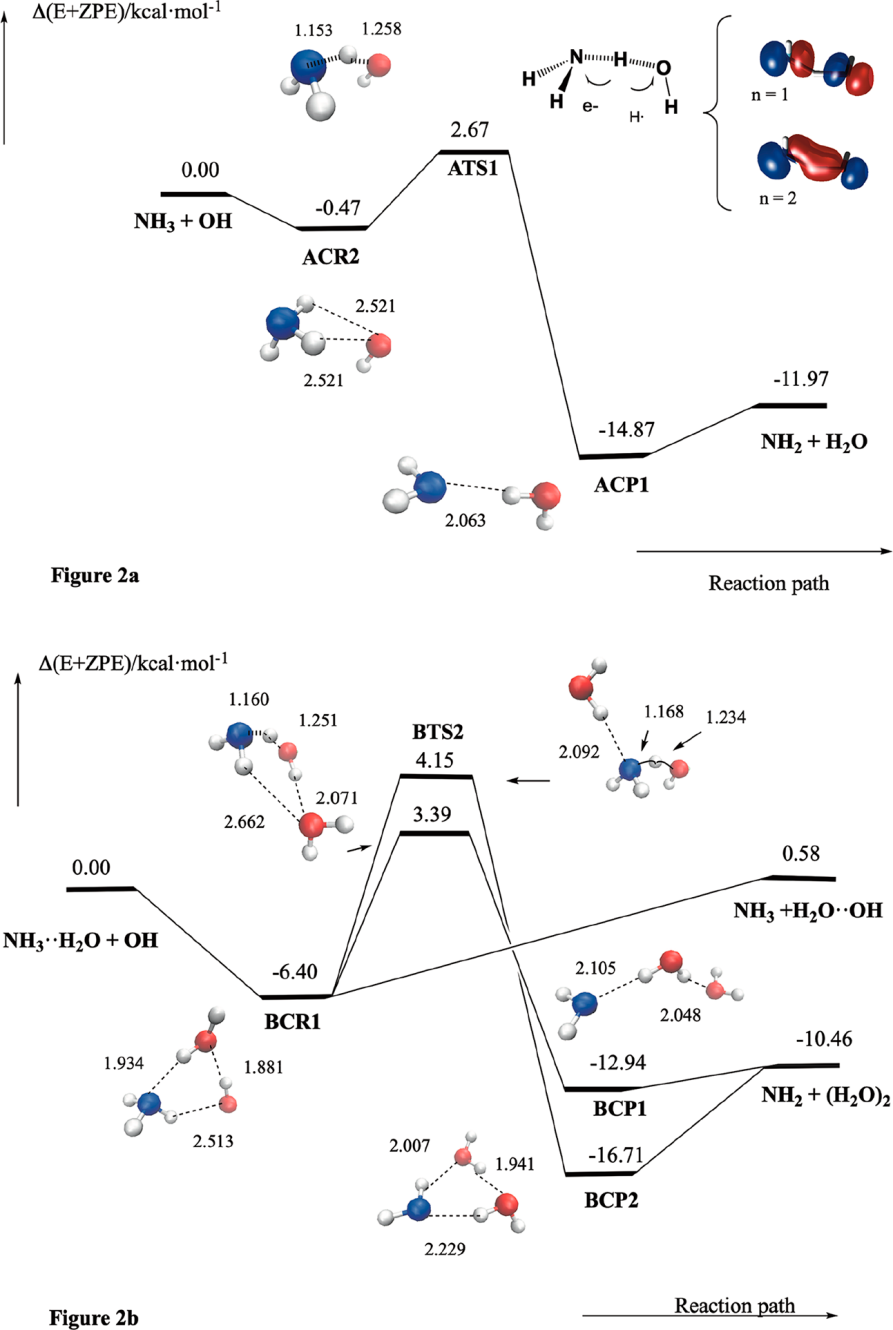
(a) Schematic potential energy surface of the NH_3_ + OH reaction, and (b) schematic potential energy surface of the NH_3_···H_2_O + OH reaction. Nitrogen atoms are in blue, oxygen atoms in red, and hydrogen atoms in white. In (a), the electronic features for **ATS1** are also plotted and the number n stands for the occupation of the corresponding orbital.

**Table 2:** Calculated Relative Energies, Energies Plus ZPE, Enthalpies and Free Energies, in kcal·mol^–1^, for the Reaction of NH_3_, and NH_3_··H_2_O, with OH^a^

compound	Δ*E*	Δ(*E*+ZPE)	Δ*H* (298 K)	Δ*G* (298 K)
**NH_3_ + OH → NH_2_ + H_2_O**				
**NH_3_ + OH**	0.00	0.00	0.00	0.00
**ACR2 (^2^A′)**	–1.43	–0.47	–0.45	4.37
**ATS1**	3.66	2.67	1.39	9.70
**ACP1 (^2^A′)**	–15.59	–14.87	–14.99	–9.71
**NH_3_ + H_2_O**	–10.49	–11.97	–11.69	–11.94
**NH_3_···H_2_O + OH → Products**				
**NH_3_···H_2_O + OH**	0.00	0.00	0.00	0.00
**BCR1**	–9.06	–6.40	–6.86	0.79
**BTS1**	4.63	3.39	2.92	10.05
**CP1**	–13.00	–12.94	–12.59	–7.84
**BTS2**	5.81	4.15	3.78	10.34
**BCP2**	–18.15	–16.71	–16.84	–9.42
**NH_2_ + (H2O)_2_**	–9.08	–10.46	–9.63	–11.98
**NH_3_ + H_2_O···OH**	0.68	0.58	0.92	–0.91

a Computed at CCSD­(T)/CBS//QCISD/6-311+G­(2df,2p). The ZPE, entropic and enthalpic corrections are computed at QCISD/6-311+G­(2df,2p).

Figure 2 shows that the reaction begins with the formation of the pre reactive complex **ACR2**, goes on through the transition state **ATS1** and forms the post-reactive complex **ACP1** which occurs before the release of the NH_3_ and H_2_O products. Please note that **ACR2** has a structure different from that of the NH_3_···OH complex (**ACR1**) discussed in the previous section. **ACR2** has *C_S_* symmetry (^2^A′), where the OH moiety lies along the molecular plane. This complex is held together by two weak hydrogen bonds formed between the oxygen atom of the hydroxyl radical and two hydrogen atoms of ammonia and has a computed binding energy of 0.47 kcal·mol^–1^ only. The elementary reaction takes place through the transition state **ATS1**, which involves the conventional hydrogen atom transfer mechanism (*hat*), consisting in the concerted breaking and forming of covalent (H_2_)­N–H and H–O­(H) bonds. The electronic features of this process are also displayed in Figure 2a, which shows that it can be described by three electrons in two orbitals (the SOMO – with occupation n = 2– and HOMO– with occupation n = 1– orbitals), where the electron density is shared between the nitrogen and oxygen atoms in which the hydrogen atom is being transferred. Our calculations predict the transition state to lie 2.67 kcal·mol^–1^ above the energy of the NH_3_ and OH reactants. At the transition state, the hydrogen being transferred is closer to the nitrogen atom (1.153Å) than to the oxygen atom (1.258Å). Figure 2 and Table 2 show that the post reactive complex **ACP1** (NH_2_···H_2_O) has C_S_ symmetry (^2^A′) and lies 14.87 kcal·mol^–1^ below the energy of the reactants. This water amidogen complex has been identified experimentally^70^ and our calculations predict a binding energy of 2.90 kcal·mol^–1^ (see Table 2), in an excellent agreement with the reported value.^70^ Table 2 also shows that the reaction energy is computed to be −11.97 kcal·mol^–1^ and the calculated reaction enthalpy at 298 K is −11.69 kcal·mol^–1^, in an excellent agreement with the experimental value of −11.40 ± 0.05 kcal·mol^–1^.^75^ The results reported in this work are also in a very good agreement with previous theoretical results from the literature.^70,71,73,74^

We investigated the effect of water vapor on the atmospheric oxidation of ammonia by considering the reaction between NH_3_···H_2_O and OH. Figure 2b shows that in the entrance channel, the reaction begins with the formation of the barrierless prereactive complex (**BCR1**), for which we have computed a binding energy of 6.40 kcal·mol^–1^. This complex has a six-member ring structure and has three moieties (NH_3_, H_2_O, and OH) that are held together by three hydrogen bonds. Please note from Figure 2 that the NH_3_···H_2_O moiety has a similar structure than the corresponding complex discussed in the previous section, but the bond length is shorter (1.934 Å versus 2.015 Å in the NH_3_···H_2_O complex). Moreover, the H_2_O···OH moiety has the same structure than the water – hydroxyl radical complex,^76^ having also a shorter bond length (1.881 Å versus 1.942 Å in the H_2_O···OH complex^76^), these results, pointing out the strength of the hydrogen bonds because the existence of cooperative effects in forming the six-member ring structure in **BCR1**. The unpaired electron lies in a plane perpendicular to the (H_2_N)-H···O-H plane.

After **BCR1** is reached, the reaction can proceed in three different ways. The first two involve the oxidation of ammonia producing amidogen radical and water dimer and take place through the elementary reaction paths with transition states **BTS1** and **BTS2**. The third one involves the barrierless decomposition of **BCR1**, producing NH_3_ and the H_2_O···OH complex. The two reaction paths producing the oxidation of ammonia (through **BTS1** and **BTS2**) take place by the conventional *hat* mechanism, involving the concerted breaking and making of the N–H and H–O bonds, as described for the naked reaction. Figure 2b shows that both transition states differ in the roles played by the water molecule. **BTS1** has recently described in the literature,^74^ and the oxygen atom of the water molecule forms one hydrogen bond with the hydrogen atom of the OH moiety and one weak hydrogen bond with one hydrogen atom of the NH_3_ moiety, so that the transition state forms a six-member ring structure. In **BTS2** one hydrogen atom of the water molecule interacts with the lone pair of the nitrogen atom in the same way as in the NH_3_···H_2_O reactant, as discussed above. Our calculations predict these transition states to lie 3.39 and 4.15 kcal·mol^–1^ above of the reactants, which is about 1.00 kcal·mol^–1^ more stable than the previously reported value.^74^ Comparing with the naked reaction (see above), we see that water vapor produces a destabilization of the transition states between 0.72-1.48 kcal·mol^–1^.

Our calculations also predict that **BCR1** can also decompose into NH_3_ + H_2_O···OH, which lie only 0.58 kcal·mol^–1^ above the NH_3_···H_2_O + OH entrance channel.

### The Reaction between NH3···HNO_3_ and OH

The most relevant results regarding the potential energy surface are displayed in Figure 3 and Table 3 and the most significant electronic features of the transition states are shown in Figure 4. The different stationary points regarding this reaction are named starting by the letter C followed by the acronym CR for the pre-reactive complexes and TS for the transition states and for a number in the reactive area, and named starting by the letter D, followed by the CR for the minima and TS for the transition states, and by a number in the product area.

**Figure 3:**
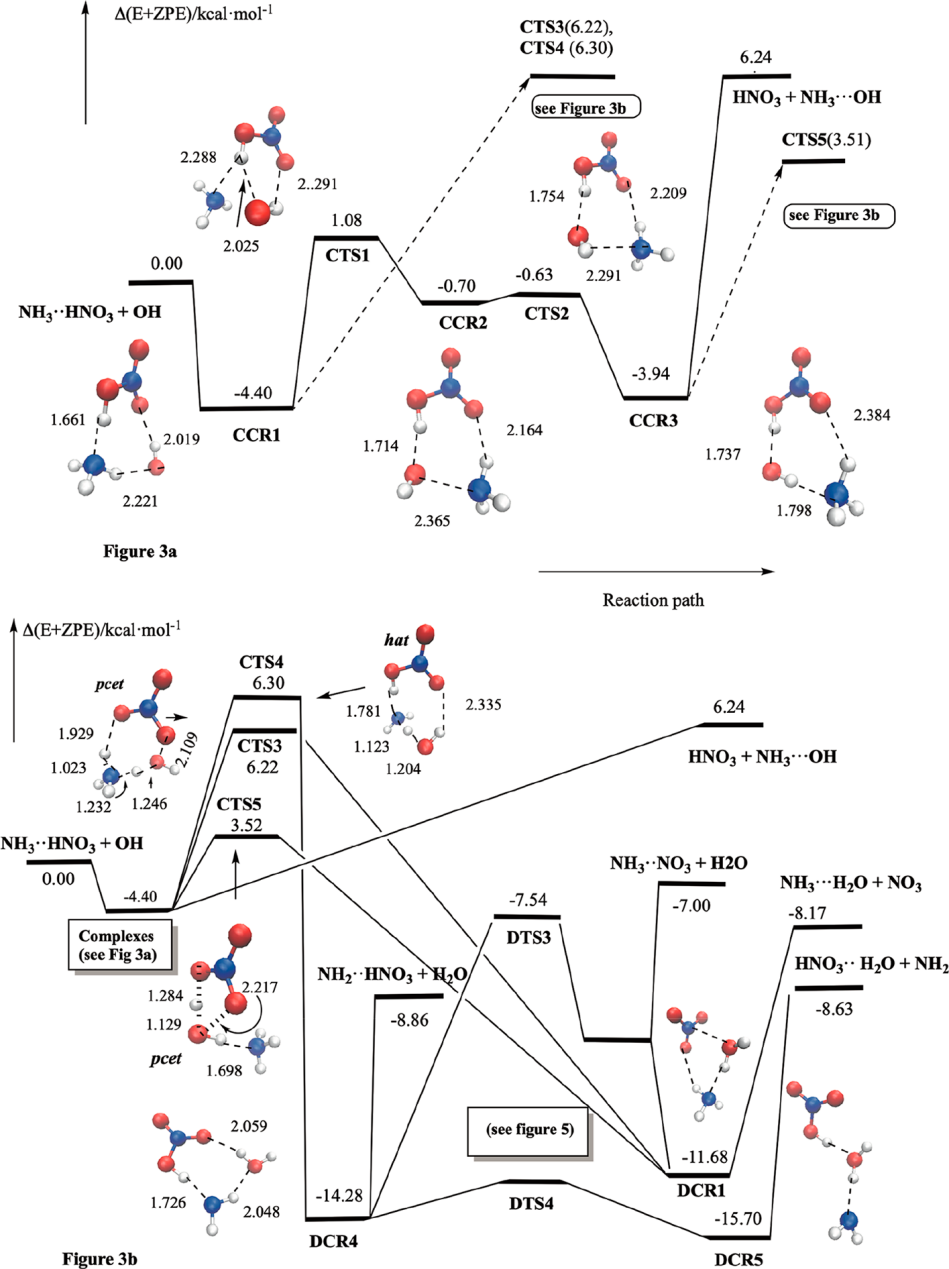
(a) Schematic potential energy surface of the pre-reactive area of the NH_3_···HNO_3_ + OH reaction, and (b) schematic potential energy surface of the reactive region. Nitrogen atoms are in blue, oxygen atoms in red and hydrogen atoms in white.

**Table 3:** Calculated Relative Energies, Energies Plus ZPE, Enthalpies and Free Energies, in kcal·mol^–1^, for the Reaction of NH_3_···HNO_3_ with OH^a^

compound	Δ*E*	Δ(*E*+ZPE)	Δ*H* (298 K)	Δ*G* (298 K)
**NH_3_···HNO_3_ + OH → Products**				
**NH_3_···HNO_3_ + OH**	0.00	0.00	0.00	0.00
**CCR1**	–6.45	–4.40	–4.97	3.36
**CTS1**	0.16	1.08	0.62	8.78
**CCR2**	–2.57	–0.70	–1.14	7.02
**CTS2**	–2.02	–0.63	–1.30	7.00
**CCR3**	–6.29	–3.94	–4.54	3.34
**CTS3**	5.38	6.22	4.59	16.10
**CTS4**	7.62	6.30	5.12	15.07
**CTS5**	3.37	3.51	2.19	12.38
**DCR1**	–11.53	–11.68	–11.07	–6.23
**DTS1**	–8.63	–10.88	–10.10	–5.73
**DCR2**	–8.82	–9.40	–8.56	–4.05
**DTS2**	–6.87	–7.09	–6.85	–0.11
**DCR3**	–10.49	–8.70	–9.35	–0.07
**DTS3**	–7.92	–7.54	–8.84	1.81
**DCR4**	–15.47	–14.28	–14.54	–6.69
**DTS4**	–9.03	–10.56	–10.52	–3.61
**DCR5**	–15.35	–15.70	–15.78	–9.63
**HNO_3_ + NH_3_···OH**	5.70	6.24	5.68	4.28
**NO_3_ + NH_3_··H_2_O**	–6.74	–8.17	–8.39	–9.47
**NO_3_···NH_3_ + H_2_O**	–4.32	–7.00	–5.86	–9.56
**HNO_3_···NH_2_ + H_2_O**	–7.57	–8.86	–8.54	–9.58
**HNO_3_···H_2_O + NH_2_**	–7.32	–8.63	–8.44	–8.95

a Computed at CCSD­(T)/CBS//QCISD/6-311+G­(2df,2p). The ZPE, entropic and enthalpic corrections are computed at QCISD/6-311+G­(2df,2p).

**Figure 4:**
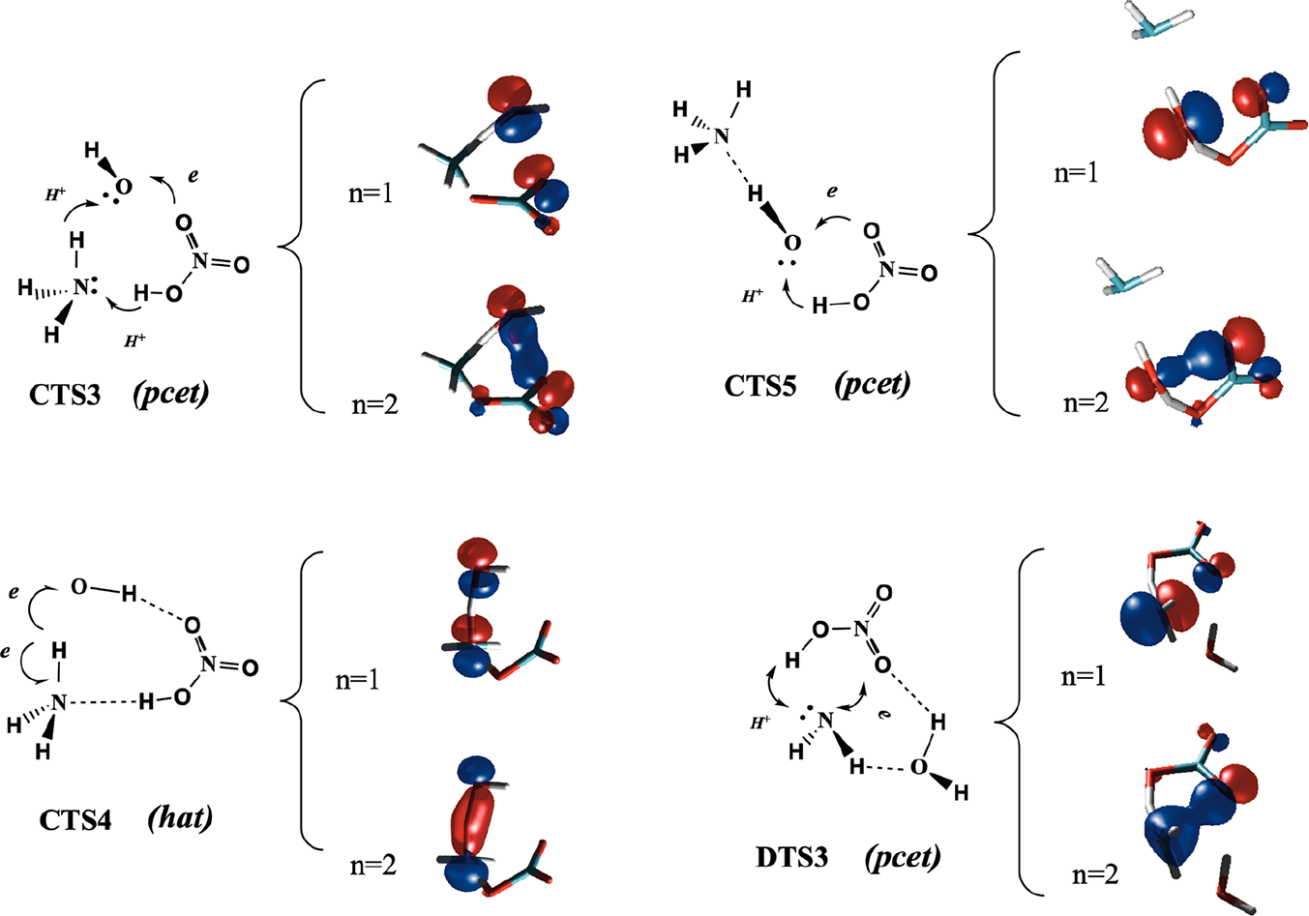
Electronic and reactive features of the transition states. The number n stands for the occupation of the corresponding orbital.

Figure 3a shows that the reaction begins with the formation of a pre-reactive complex **CCR1**, for which we have computed a binding energy of 4.40 kcal·mol^–1^. This complex has an eight-member ring structure, which is formed by three moieties HNO_3_, NH_3_, and OH that are held together by three hydrogen bonds; the first one between the hydrogen atom of nitric acid and the lone pair of ammonia (*d*_HN_ = 1.661 Å), the second one between one hydrogen atom of ammonia and the oxygen atom of hydroxyl radical (*d*_OH_ = 2.221 Å), and the third one between hydrogen atom of OH and one oxygen atom of (HO)­NO_2_ (2.019 Å). A closer look at Figure 3a shows that this complex retains the structure of the HNO_3_···NH_3_ reactant (see Figure 1) in which OH has been added closing the ring. A very interesting feature of this complex is the very short distance computed for the N­(O_2_)­OH···NH_3_ hydrogen bond length (1.661 Å), compared with the 1.718 Å computed for the N­(O_2_)­OH···NH_3_ bond length, which indicates a strengthening of this interaction. At a first glance, the structure of **CCR1** seems to indicate that ammonia hampers nitric acid to be oxidized by hydroxyl radical, which could only attack one of the hydrogen atoms of the NH_3_ moiety. However, a reorganization of this pre-reactive complex is possible and takes place through the transition state **CTS1**, the intermediate **CCR2**, the transition state **CTS2** and finally the complex **CCR3**. Figure 3a shows that this reorganization produces a structural change in the NH_3_···OH moiety so that in **CCR3** the OH radical faces the hydrogen atom of HNO_3_, and the hydroxyl radical is able to oxidize nitric acid. In **CCR3**, the three moieties HNO_3_, OH, and NH_3_ are held together by three hydrogen bonds, one between the hydrogen atom of the acid and the oxygen atom of the radical (*d*_HO_ = 1.737 Å), one between the hydrogen atom of the OH moiety and the lone pair over the nitrogen atom of ammonia (*d*_HN_ = 1.798 Å), and the third between one hydrogen atom of NH_3_ and one oxygen atom of HNO_3_ (*d*_OH_ = 2.384 Å). Our calculations predict this complex to lies 3.94 kcal·mol^–1^ below the energy of the separate reactants (NH_3_···HNO_3_ + OH) and its only 0.46 kcal·mol^–1^ less stable than **CCR1**.

Beginning with the pre-reactive complex **CCR1**, the reaction can go on through two different reaction paths (via **CTS3** and **CTS4**, see Figure 3a,b) involving two different processes. The reaction through **CTS3** leads to the direct oxidation of the nitric acid by hydroxyl radical, producing NO_3_ + NH_3_···H_2_O. This is an unexpected mechanism because, on the reactants side, ammonia seems to hamper the direct interaction between nitric acid and hydroxyl radical. From an electronic point of view, Figure 4 shows that this process is described by three electrons in three orbitals where one oxygen atom of the nitric acid moiety faces the oxygen atom of the OH moiety, which allows one electron to be transferred from the acid to the radical and simultaneously, one proton of HNO_3_ moves to ammonia, and one proton from ammonia is transferred to the oxygen atom of the hydroxyl radical. This is a proton coupled electron transfer mechanism (*pcet*), and our calculations predict **CTS3** to lie 6.22 kcal·mol^–1^ above the energy of the NH_3_···HNO_3_ + OH separate reactants.

The reaction occurring via **CTS4** involves the expected oxidation of the ammonia moiety by hydroxyl radical leading to the formation of HNO_3_ + NH_2_ + H_2_O product The **CTS4** transition state is computed to lie 6.30 kcal·mol^–1^ above the energy of the separate reactants and his structure has an eight-membered ring structure similar to the pre-reactive **CCR1** complex. Figure 4 shows that the electronic features of this transition state correspond to the a concerted breaking and making of the (H_2_)­N···H···O­(H) bonds and corresponds to a conventional *hat* mechanism, as described above for the NH3 + OH and for the NH_3_···H_2_O + OH reactions. In this case, the electronic density describing this process (three electrons in three orbitals) is localized over the atoms involved in the hydrogen transfer process (the N atom of the ammonia moiety and the oxygen atom of the OH radical moiety).

Starting from **CCR3**, the reaction can continue in two different ways. The first one goes through **CTS5** and produces the oxidation of nitric acid into NO_3_, and our calculations predict this transition state to lie 3.51 kcal·mol^–1^ above the energy of the separate reactants, about 2.7 kcal·mol^–1^ more stable than the processes via **CTS3** and **CTS4**. Figure 4 shows that this transition state also follows a *pcet* mechanism. At the transition state, the different moieties approach each other in such a way that the lone pair over the oxygen atom of the NO group faces the radical of the hydroxyl moiety, whereas the lone pair of the OH group is directed toward the hydrogen atom of nitric acid, so that there is a transfer of one electron from one oxygen atom of nitric acid to one oxygen of the hydroxyl moiety, and simultaneously, the proton of nitric acid is transferred to the OH group. The ammonia moiety is linked to the hydrogen atom of the OH group by a hydrogen bond, which does not participate directly in the reaction. It is also worth mentioning that the electronic and geometric features of **CTS5** are very similar to those described for the transition states involving in the gas phase oxidation of several acids by hydroxyl and Cl, ClO, and NH_2_ radicals involving *pcet* reaction mechanisms, and in particular with the reaction of HNO_3_···H_2_O with OH.^14,15,22,77–87^

Regarding the fate of the elementary processes via **CTS3**, **CTS4**, and **CTS5**, Figure 3b shows a very complex potential energy surface in the product region, which connects the results of the oxidation of nitric acid moiety, namely formation of the NO_3_ radical, with the results of the oxidation of the ammonia moiety, namely formation of the NH_2_ radical. Our calculations predict the different products to be between 7.00 and 8.86 kcal·mol^–1^ more stable than the NH_3_···HNO_3_ + OH separate reactants and to be formed with an excess energy of about 12 kcal·mol^–1^. Therefore, it is expected that the oxidation of both HNO_3_ and NH_3_ moieties will occur. A more detailed discussion of this area of the potential energy surface will be discussed in the next section.

Finally, a closer look at the structure of **CCR3** shows that it can dissociate into HNO_3_ + NH_3_···OH in a barrierless process through the breakdown of the two (H)­O···HNO_3_ and HON­(O)­O···H­(NH_2_) hydrogen bonds. Figure 3a and Table 3 show that this reaction is predicted to be endothermic by 6.24 kcal·mol^–1^.

### The Reactions of NH_3_···H_2_O with NO_3_ and HNO_3_···H_2_O + NH_2_

In the previous sections, we have pointed out that the oxidation of NH_3_···HNO_3_ by OH radical leads to a complex potential energy surface in the product area where either NO_3_ + NH_3_ + H_2_O or HNO_3_ + H_2_O + NH_2_ products can be formed, and the corresponding potential energy surface has been schematized in Figure 5. In Table 4, we also collected the relative energies of both reactions.

**Figure 5:**
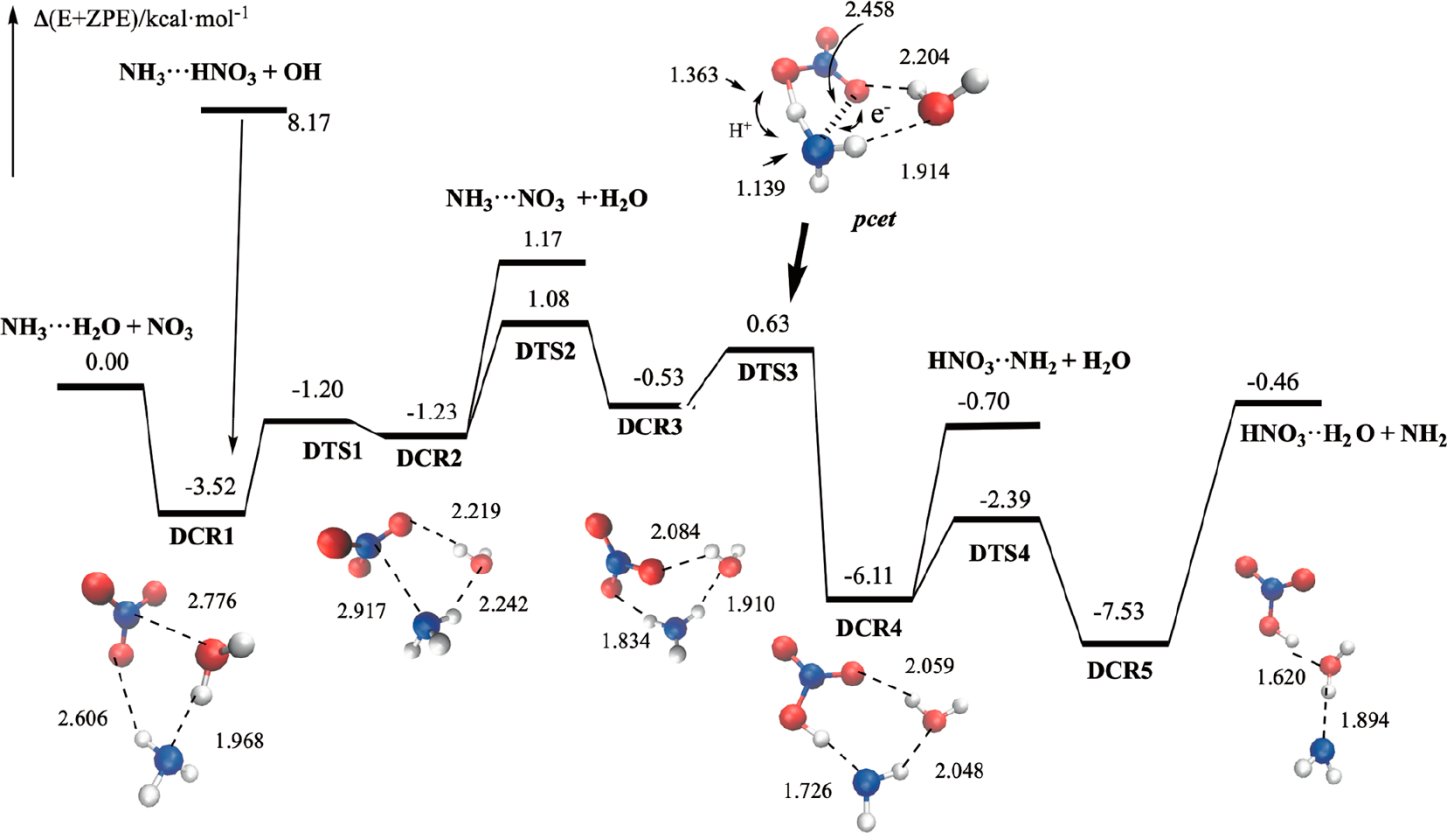
Schematic potential energy surface of the NH_3_··H_2_O + NO_3_ and HNO_3_···H_2_O + NH_2_ reactions. Nitrogen atoms are in blue, oxygen atoms in red, and hydrogen atoms in white.

**Table 4:** Calculated Relative Energies, Energies Plus ZPE, Enthalpies and Free Energies, in kcal·mol^–1^, for the Reaction of HNO_3_··H_2_O + NH_2_, and NO_3_ + NH_3_···H_2_O^a^

compound	Δ*E*	Δ(*E*+ZPE)	Δ*H* (298 K)	Δ*G* (298 K)
**HNO_3_···H_2_O + NH_2_ → NO_3_ + (NH_3_···H_2_O)**				
**HNO_3_···H_2_O + NH_2_**	0.00	0.00	0.00	0.00
**DCR5**	–8.04	–7.07	–7.34	–0.68
**DTS4**	–1.71	–1.93	–2.08	5.34
**DCR4**	–8.16	–5.65	–6.09	2.26
**DTS3**	–0.60	1.09	–0.40	10.76
**DCR3**	–3.18	–0.07	–0.90	8.87
**DTS2**	0.44	1.54	1.60	8.84
**DCR2**	–1.51	–0.77	–0.11	4.90
**DTS1**	–1.32	–2.26	–1.66	3.22
**DCR1**	–4.21	–3.06	–2.63	2.72
**NH_2_···HNO_3_ + H_2_O**	–0.25	–0.24	–0.10	–0.63
**NO_3_ + NH_3_···H_2_O**	0.57	0.46	0.06	–0.53
**NH_3_···NO_3_ + H_2_O**	3.00	1.63	2.59	–0.61
**NO_3_ + NH_3_···H_2_O → NH_2_···HNO_3_ + H_2_O**				
**NH_3_···H_2_O + NO_3_**	0.00	0.00	0.00	0.00
**DCR1**	–4.78	–3.52	–2.69	3.25
**DTS1**	–1.89	–2.72	–1.72	3.74
**DCR2**	–2.08	–1.23	–0.17	5.43
**DTS2**	–0.13	1.08	1.54	9.36
**DCR3**	–3.75	–0.53	–0.96	9.40
**DTS3**	–1.18	0.63	–0.46	11.28
**DCR4**	–8.73	–6.11	–6.15	2.78
**DTS4**	–2.28	–2.39	–2.14	5.87
**DCR5**	–8.61	–7.53	–7.40	–0.16
**NH_2_···HNO_3_ + H_2_O**	–0.83	–0.70	–0.16	–0.11
**NH_3_···NO_3_ + H_2_O**	2.42	1.17	2.53	–0.08
**HNO_3_···H_2_O + NH_2_**	–0.57	–0.46	–0.06	0.53

a Computed at CCSD­(T)/CBS//QCISD/6-311+G­(2df,2p). The ZPE, entropic and enthalpic corrections are computed at QCISD/6-311+G­(2df,2p).

From a theoretical point of view. it is interesting to remind here the well-known doublet instability phenomenon of nitrate radical and the difficulties of predicting accurate relative energies.^15,88–92^ The same situation occurs for the complexes of nitrate radical with different species, as investigated in this work for the pre-reactive area of the NH_3_···H_2_O + NO_3_ reaction, and in order to avoid this issue, we have studied the corresponding stationary points employing the QCISD theory, which is proven to be a good approach for this systems.^15^

A closer look at Figure 5 shows that the potential energy surface describes two different reactions, namely, the oxidation of ammonia by nitrate radical and the oxidation of nitric acid by amidogen radical, both assisted by a single water molecule. These two reactions are connected so that the products of one reaction are the reactants of the opposite and the relative reactants/products differ in only 0.46 kcal·mol^–1^. These processes are important for atmospheric purposes since the first one may operate at nighttime and the second one may operate at daytime. In fact, the naked reactions were already reported, a new atmospheric catalytic cycle were proposed,^14,15^ and in this work the effect of water is analyzed.

Again, and in a similar manner as described in the previous sections, we have found that both reactions begin with the existence of several pre-reactive complexes that reorganize before the reactive steps takes place. Figure 5 shows that, starting from the NH_3_···H_2_O + NO_3_ reactants, the reaction goes through the **DCR1**, **DCR2**, and **DCR3** pre-reactive complexes before the **DTS3** reactive transition states and the formation of the products, whereas starting from HNO_3_···H_2_O reactants, the reaction goes through the **DCR5** and **DCR4** pre-reactive complexes before the **DTS3** reactive transition state and the formation of the products. Figure 5 and Figure 6 show that **DTS3** involve a *pcet* mechanism and the transfer of one electron and one proton involved in this process depends on which reaction occurs. In the NO_3_ + NH_3_···H_2_O reaction, the electron jumps form the lone pair of the NH_3_ moiety to one oxygen atom of the NO_3_ radical and, on the other side of the molecules, one proton from the NH_3_ moiety is transferred to another oxygen atom of the NO_3_ moiety.

**Figure 6:**
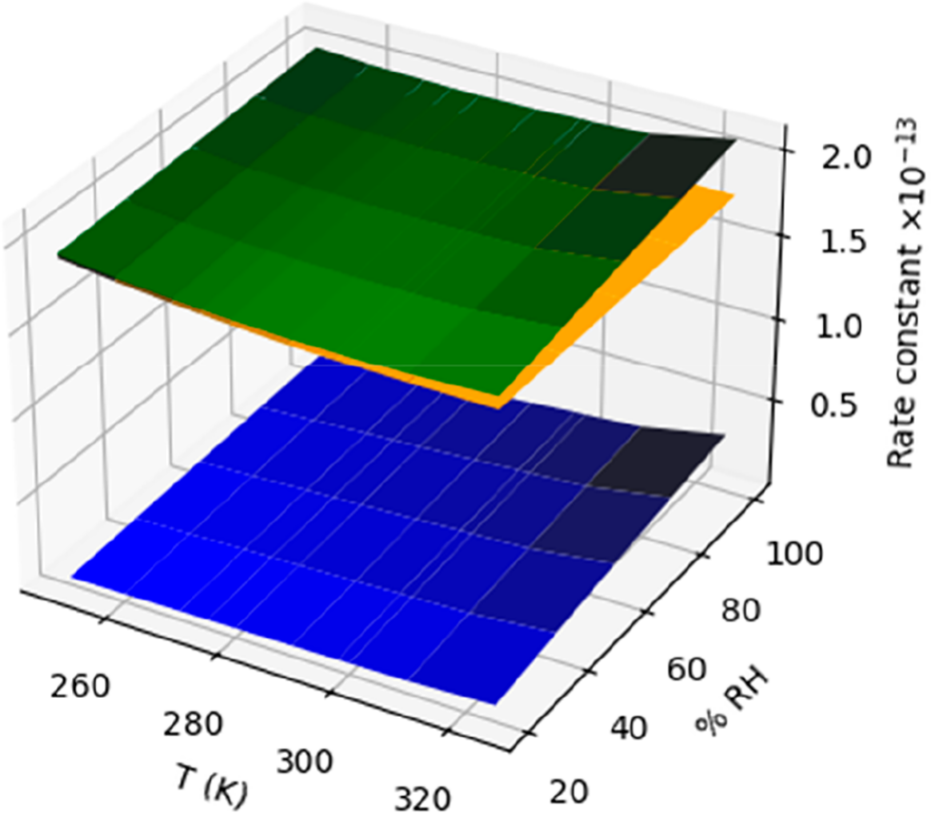
Effect of water vapor on the rate constant (in cm^3^·molecule^–1^·s^–1^) of the reaction of nitric acid with amidogen radical. The rate constants of the HNO_3_···H_2_O + NH_2_ are in blue, those of the naked reaction is in orange, and the corresponding values of the whole effect (HNO_3_ + H_2_O + NH_2_ reactions) are in green.

Starting from the HNO_3_···H_2_O + NH_2_ radical, the electron is transferred from one oxygen atom of the acid to the nitrogen atom of the amidogen moiety and the proton of the acid is transferred to the NH_2_ radical. The water molecule interacts via a hydrogen bond with the oxygen of the (H)­NO_3_ and with one hydrogen of the (H)­NH_2_ part. The structure and electronic features are very similar as described above for the **CTS5** transition state described in the previous section and for the transition state of the HNO_3_ + NH_2_ naked reaction.^14,15^ Figure 5 and Table 4 show that **DTS3** lies energetically 0.63 kcal·mol^–1^ above the NO_3_ + NH_3_···H_2_O reactants and 1.09 kcal·mol^–1^ above the HNO_3_···H_2_O + NH_2_ reactants, and it is the limiting step of both reactions (11.28 or 10.76 kcal·mol^–1^ when free energies are taken into account)

### Reaction Kinetics

In the previous sections, we have pointed out that the reactions investigated in this work show different complexity. The naked reactions, namely the reaction of ammonia with hydroxyl radical, the reactions of nitric acid with amidogen radical, or the reaction of ammonia with nitrate radical, follow the scheme of reaction 8, that is, they begin with the formation of a pre-reactive complex, which takes place before the transition state leading to the formation of the products.

8 $$A+B\overset{k_{1}}{\underset{k_{-1}}{⇌}}\mathrm{pre‐reactive complexes}\overset{k_{2}}{\to }\mathrm{products}$$

In these cases, the rate constant (*k_I_*) has been calculated according to equation 9,

9 $$k_{I}=\frac{k_{1}}{k_{-1}}k_{2}=K_{\mathrm{eq}}K_{2}$$

Where we have considered that the pre-reactive complexes are in equilibrium with the reactants, *K*_eq_ is the equilibrium constant of the pre-reactive complex and *k*_2_ is the rate constant of the unimolecular reaction between the pre-reactive complex and the reaction products. These equilibrium constants have been calculated according to equation 10

10 $$K_{\mathrm{eq}}=.\frac{Q_{\mathrm{complex}}}{Q_{A}Q_{B}}.e^{\frac{-\left(E_{C}-E_{R}\right)}{RT}}$$

where the various *Q* denote the partition functions of the reactants A and B, and the pre-reactive complex; and *E*_R_ and *E*_C_ are the total energies of the reactants and the pre-reactive complex respectively. *k*_2_ has been computed employing the canonical variational transition state theory (CVTST) according to equation 11.^93,94^

$$k^{CVT}=k\frac{k_{B}T}{h}\frac{Q_{\mathrm{GT}}(S^{*})}{Q_{\mathrm{complex}}}e^{\frac{-V(S^{*})}{k_{B}T}}$$ 11

Where s* is the free energy maximum along the reaction path at temperature *T*, *Q*_complex_ is the partition function of the pre-reactive complex, *Q*(s*) is the generalized transition state partition function, *V*(s*) is the potential energy, and *κ* is the tunneling parameter computed with the small curvature approach. The Polyrate program has been used for these calculations.^95^

The reaction between HNO_3_···NH_3_ + OH (reaction 4) and the reactions involving a water molecule (reactions 5, 6, and 7) is more intricate since there are several connected hydrogen-bond complexes occurring before the reactive transition state. In these cases, we have calculated the rate constants by numerical integration of the rate equations according the schemes and kinetic equations reported in the supplementary material (Figures S1, S2, and S3 and Tables S13–S18) in a similar manner as we did in a previous work.^96^ The integration of the kinetic equations was done with inhouse scripts using the Scipy Python library which implements a version of the odeint routine.^97,98^ The step size of the first step in the numerical integration we carried out is 1 × 10^–14^. For these calculations, we have considered conventional transition state theory for the elementary steps connecting the hydrogen bond complexes and variational transition state theory for the elementary steps involving the reactive transition state We have also calculated the tunneling parameter with the small curvature approach. More details of these calculations are reported in the Supporting information.

Our results regarding the oxidation of ammonia by hydroxyl radical (reaction 1) (Table S4, which shows that at 300 K our computed rate constant is 1.27 × 10^–13^ cm^3^·molecule^–1^·s^–1^ and our calculated Arrhenius equation in the range of 250–320 T is 4.11 × 10^–12^·e^(‑1042.08/T)^, in very good agreement of the calculated and experimental value of 1.6 × 10^–13^ cm^3^·molecule^–1^·s^–1^,^73,99,100^ and 1.03 × 10^–13^ cm^3^·molecule^–1^·s^–1^,^74^ with an Arrhenius equation of 3.5 × 10^–13^·e­(^−925/T^).^99^ regarding the reaction involving one water molecule (reaction 7), we have calculated rate constants ranging from 3.14 × 10^–17^ cm^3^·molecule^–1^·s^–1^ at 300 K and 20% of relative humidity (RH), to 3.57 × 10^–16^ cm^3^·molecule^–1^·s^–1^ at 320 K and 100% of RH (see Tables S3 and S4) so that the contribution of the humidity to the rate constants is negligible in agreement with the results reported in the literature.

The reaction NH_3_···HNO_3_ + OH involves oxidation of either the ammonia moiety or the nitric acid moiety. We have calculated a rate constant of 6.50 × 10^–16^ cm^3^·molecule^–1^·s^–1^, at 298 K, and an Arrhenius equation 5.80 × 10^–15^·e^(‑1.29/*RT*)^, as shown in Figure S2 and Table S5. In agreement with the atmospheric importance of the NH_3_···HNO_3_ complex reported in the literature,^23^ we suggest that this reaction plays an important role in the atmosphere. At this point, it is also worth comparing the rate constant of the NH_3_···HNO_3_ + OH reaction with that of the HNO_3_···H_2_O + OH reaction as both reactions have similar geometrical and electronic structure in the transition state. The calculated rate constant for the water assisted oxidation of nitric acid by hydroxyl radical is 1.6 × 10^–16^ cm^3^·molecule^–1^·s^–1^, at 298 K^22^ in line with the corresponding value of the present reaction.

The full results of the effect of water vapor on the reaction of nitric acid with the amidogen radical are displayed in Tables S6 and S7 of the Supporting Information, according to the kinetic scheme of Figure S3. The results are plotted in Figure 6, which shows the effect of water vapor in the reaction. The calculated values of the rate constant of the naked reaction were already reported in a previous work,^14^ and range between 1.91 × 10^–13^ cm^3^·molecule^–1^·s^–1^, at 250 K and 1.77 × 10^–13^ cm^3^·molecule^–1^·s^–1^ at 325 K (orange surface in Figure 6), with and the calculated Arrhenius equation is 1.35 × 10^–13^·e­(^0.17/RT^).

Our computed values of the rate constants of the reaction of HNO_3_···H_2_O with NH_2_ range between 1.90 × 10^–15^ cm^3^·molecule^–1^·s^–1^, at 250 K and 20% of RH, 1.97 × 10^–14^ cm^3^·molecule^–1^·s^–1^, at 298 K and 100% of RH, and 3.26 × 10^–14^ cm^3^·molecule^–1^·s^–1^, at 325 K and 100% of RH (see Table S6). These values indicate that the impact of water vapor on the reaction of nitric acid with the amidogen radical is small and only significant at very hot and humid conditions (green surface in Figure 6 and Table S7), with a total rate value of 2.10 × 10^–13^ cm^3^·molecule^–1^·s^–1^, at 325 K and 100% RH. This implies an increase of 18% of the rate constant with respect to the value of the naked reaction. For other conditions, see Table S8.

Finally, our results regarding the effect of water on the rate constants of the NO_3_ + NH_3_ reaction are displayed in Figure 7 and in Tables S10–S12. We have calculated that value of the rate constants of the naked reaction with values ranging between 6.02 × 10^–17^ cm^3^·molecule^–1^·s^–1^, at 250 K and 1.99 × 10^–16^ cm^3^·molecule^–1^·s^–1^, at 325 K (in orange in Figure 7), which are roughly two times greater than the values reported previously,^14^ and with a calculated Arrhenius equation of 1.05 × 10^–14^·e^(−2.57/*RT*)^.

**Figure 7:**
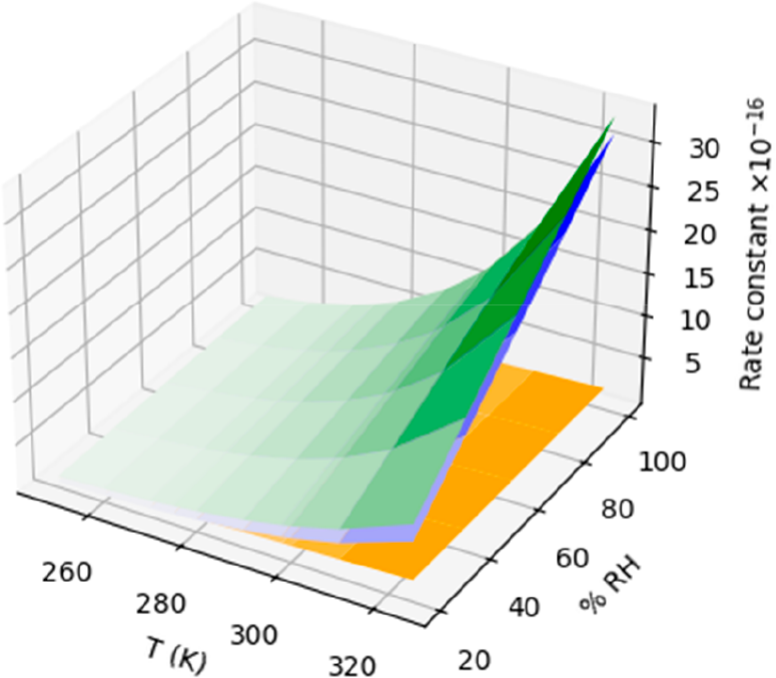
Effect of water vapor on the rate constant of the reaction of nitric acid with the amidogen radical. The rate constants of the NO_3_···H_2_O + NH_3_ are in blue, those of the bare reaction are in orange, and the corresponding values of the whole effect (NO_3_ + H_2_O + NH_3_ reactions) are in green.

For the reaction of NH_3_···H_2_O with NO_3_, our calculated rate constants range from 1.49 × 10^–17^ cm^3^·molecule^–1^·s^–1^ at 250 K and 20% of RH, to 3.14 × 10^–15^ cm^3^·molecule^–1^·s^–1^ at 325 K and 100% of RH (orange surface in Figure 7 and Table S10). In this case, we predict an important impact of water vapor on the reaction of ammonia with nitrate radical, with values ranging between 7.51 × 10^–17^ cm^3^·molecule^–1^·s^–1^, at 250 K and 20% of RH, 1.16 × 10^–15^ cm^3^·molecule^–1^·s^–1^, at 298 K and 100% of RH, and 3.34 × 10^–15^ cm^3^·molecule^–1^·s^–1^, at 325 K and 100% of RH (green surface in Figure 7) for the whole reaction (NH_3_ + NO_3_ + H_2_O). In contrast to the previous reaction, we predict an increase of the rate constants from 25% at 250 K and 20% RH up to 1580% at 325 K and 100% RH with respect to the values of the naked reaction (Tables S11 and S12).

## Conclusions

In this work, we have reported the results of a theoretical investigation on the atmospheric oxidation of ammonia and ammonia complexed with nitric acid with hydroxyl radical and the oxidation of nitric acid by amidogen radical and ammonia by nitrate radical, both taking into account the effect of water vapor.

From an electronic point of view, the oxidation of ammonia by a hydroxyl radical follows a hydrogen atom transfer mechanism (*hat*), but the key steps on the NH_3_···HNO_3_ + OH, HNO_3_···H_2_O + NH_2_, and NH_3_···H_2_O + NO_3_ reactions are described by a proton coupled electron transfer mechanism (*pcet*).

The calculated rate constant for the reaction of ammonia by hydroxyl radical is 1.24 × 10^–13^ cm^3^·molecule^–1^·s^–1^ at 298 K, and the effect of water vapor on this reaction is negligible.

For the reaction of HNO_3_···NH_3_ + OH, we have calculated a rate constant of 6.50 × 10^–16^ cm^3^·molecule^–1^·s^–1^ at 298 K. This reaction has a very complex potential energy surface which allows oxidation of either the NH_3_ or the HNO_3_ moieties. The potential energy surface of the exit channel is also very intricate and connects the reaction involving the oxidation of ammonia by nitrate radical with the oxidation of nitric acid by amidogen radical, both assisted by a water molecule.

The effect of water vapor on the oxidation of nitric acid by the amidogen radical is small but not negligible. For the whole reaction (HNO_3_ + NH_2_ + H_2_O), we have calculated a rate constant of 1.98 × 10^–13^ cm^3^·molecule^–1^·s^–1^, at 298 K and 100% of RH, which is an 11% greater than the naked reaction, and this percentage increases up to 18% at 325K and 100% of RH. For the reverse reaction, namely the oxidation of ammonia by nitrate radical, the effect of water vapor is much more important. At 298 K and 100% of RH we have calculated a rate constant of 1.16 × 10^–15^ cm^3^·molecule^–1^·s^–1^ for the whole reactions (NH_3_ + NO_3_ + H_2_O), which is 751% greater than the value of the naked reaction at 298 K. This percentage increases up to 1580% at very hot and humid conditions (325 K and 100% of RH). Thus, the effect of water vapor on the oxidation of nitric acid by the amidogen radical, which operates in the daytime, is significant and very important for the oxidation of ammonia by the nitrate radical, operating in nighttime,

## Supplementary Material



## Notes

Absolute energies, equilibrium constants of the complexes, schemes of the reactions and kinetical equations for which numerical integration of the rate constants has been done, tables of all rate constants and Cartesian Coordinates of all located stationary points (PDF
