# Supplementary material for: Atmospheric Oxidation of NH3, HNO3 and NH3···HNO3 by OH, NH2, and NO3 Radicals. The Effect of Water Vapor
Source: J Phys Chem A. 2026 Jan 7;130(3):623–35. doi: 10.1021/acs.jpca.5c06826 (PMC12833860; doi:10.1021/acs.jpca.5c06826)
Supplement: Supplementary file 1 [file jp5c06826_si_001.pdf]

Atmospheric Oxidation of NH_3, HNO_3 and $NH_3 \cdots HNO_3$ by OH, NH2, and NO3 Radicals. The Effect of Water Vapor*

Josep M. Anglada,* Ramon Crehuet*

Institute for Advanced Chemistry of Catalonia (IQAC) – CSIC, c/Jordi Girona 18-26, E-08034 Barcelona, Spain. E-mail: anglada@iqac.csic.es, rcsqtc@iqac.csic.es

(Supplementary material)

	E(QCISD)[a]	E(HF)[b]	E(CCSD(T)) [b]	E(HF) [b]	E(CCSD(T) [b]
	6-311+G(2df,2p)	aug-cc-pVTZ	aug-cc-pVTZ	aug-cc-pVQZ	aug-cc-pVQZ
H_2O	-76.32100	-76.06063	-76.34228	-76.06602	-76.36358
NH_2	-55.78411	-55.58807	-55.79983	-55.59140	-55.81308
$H_2O \cdot\cdot OH$	-151.95883	-151.48958	-151.99721	-151.49993	-152.03731
$(H_2O)x_2$	-152.65025	-152.12701	-152.69286	-152.13776	-152.73527
OH	-75.62855	-75.42167	-75.64557	-75.42667	-75.66449
NH_3	-56.46136	-56.22028	-56.48055	-56.22392	-56.49573
NO_3	-279.78382	-278.87997	-279.86870	-278.89904	-279.94086
HNO_3	-280.46591	-279.56536	-280.54459	-279.58440	-280.61839
$NH_3 \cdot\cdot OH$	-132.10194	-131.65129	-132.13863	-131.65983	-132.17261
NH_3-H_2O	-132.79241	-132.28802	-132.83332	-132.29699	-132.86964
NO_3-NH_3	-336.25171	-335.10417	-336.35609	-335.12679	-336.44314
$HNO_3 \cdot\cdot NH_3$	-336.94812	-335.80317	-337.04691	-335.82558	-337.13562
$HNO_3 \cdot\cdot H_2O$	-356.80312	-355.63962	-356.90361	-355.66387	-356.99844

ACR2	-132.09157	-131.64071	-132.12847	-131.64930	-132.16252
ATS1	-132.07789	-131.59761	-132.12010	-131.60603	-132.15425
ACP1	-132.11323	-131.65446	-132.15042	-131.66315	-132.18485
BCR1	-208.43492	-207.71940	-208.49362	-207.73324	-208.54867
BTS1	-208.40782	-207.66430	-208.47145	-207.67806	-208.52668
BCP1	-208.44060	-207.71911	-208.49921	-207.73317	-208.55471
BTS2	-208.40588	-207.66291	-208.46964	-207.67666	-208.52482
BCP2	-208.44828	-207.72423	-208.50727	-207.73824	-208.56284
CCR1	-412.58679	-411.23163	-412.70329	-411.25895	-412.81059
CTS1	-412.57600	-411.21807	-412.69263	-411.24548	-412.80002
CCR2	-412.57940	-411.21441	-412.69675	-411.24180	-412.80428
CTS2	-412.57928	-411.21917	-412.69597	-411.24660	-412.80345
CCR3	-412.35265 [b]	-411.23107	-412.70322	-411.25838	-412.81041
CTS5	-412.56676	-411.19075	-412.68752	-411.21776	-412.79481
CTS4	-412.55915	-411.17571	-412.68079	-411.20288	-412.78810
CTS3	-412.56180	-411.17440	-412.68392	-411.20156	-412.79148
DCR1	-412.58414	-411.17111	-412.71043	-411.19898	-412.81841
DTS1	-413.38500	-411.16565	-412.70616	-411.19347	-412.81393
DCR2	-412.58001	-411.16709	-412.70622	-411.19501	-412.81416
DTS2	-411.19993	-411.21645	-412.70366	-411.24425	-412.81125
DCR3	-412.58844	-411.20736	-412.70781	-411.23488	-412.81627
DTS3	-412.58517	-411.21342	-412.70402	-411.24075	-412.81225
DCR4	-412.60025	-411.23736	-412.71665	-411.26488	-412.82459
DTS4	-411.21059	-411.22764	-412.70644	-411.25523	-412.81436

DTS4	-413.37935 [c]	-411.22764	-412.70644	-411.25523	-412.81436
DCR5	-413.39162 [c]	-411.22764	-412.70644	-411.25523	-412.81436

a) Equilibrium constants of several complexes

Table S2: Calculated equilibrium constants (K_{eq} in $cm^3 \cdot molecule^{-1}$) for the formation of the complexes between NH_3 with OH, with H_2O, and with HNO_3, respectively and between HNO_3 with H_2O.

T (K)	$NH_3 \cdots OH$	$NH_3 \cdots H_2O$	$NH_3 \cdots HNO_3$	$HNO_3 \cdots H_2O$
**250**	$2.94 \cdot 10^{-20}$	$9.91 \cdot 10^{-21}$	$7.82 \cdot 10^{-16}$	$2.30 \cdot 10^{-18}$
**260**	$1.91 \cdot 10^{-20}$	$6.90 \cdot 10^{-21}$	$3.26 \cdot 10^{-16}$	$1.23 \cdot 10^{-18}$
**270**	$1.29 \cdot 10^{-20}$	$4.94 \cdot 10^{-21}$	$1.46 \cdot 10^{-16}$	$6.94 \cdot 10^{-19}$
**280**	$8.92 \cdot 10^{-21}$	$3.63 \cdot 10^{-21}$	$6.89 \cdot 10^{-17}$	$4.08 \cdot 10^{-19}$
**290**	$6.34 \cdot 10^{-21}$	$2.72 \cdot 10^{-21}$	$3.44 \cdot 10^{-17}$	$2.49 \cdot 10^{-19}$
**298**	$4.92 \cdot 10^{-21}$	$2.20 \cdot 10^{-21}$	$2.05 \cdot 10^{-17}$	$1.72 \cdot 10^{-19}$
**300**	$4.62 \cdot 10^{-21}$	$2.09 \cdot 10^{-21}$	$1.81 \cdot 10^{-17}$	$1.57 \cdot 10^{-19}$
**310**	$3.44 \cdot 10^{-21}$	$1.63 \cdot 10^{-21}$	$9.90 \cdot 10^{-18}$	$1.03 \cdot 10^{-19}$
**320**	$2.62 \cdot 10^{-21}$	$1.29 \cdot 10^{-21}$	$5.64 \cdot 10^{-18}$	$5.73 \cdot 10^{-20}$

b) Reaction of NH_3 + OH with and without H_2O.

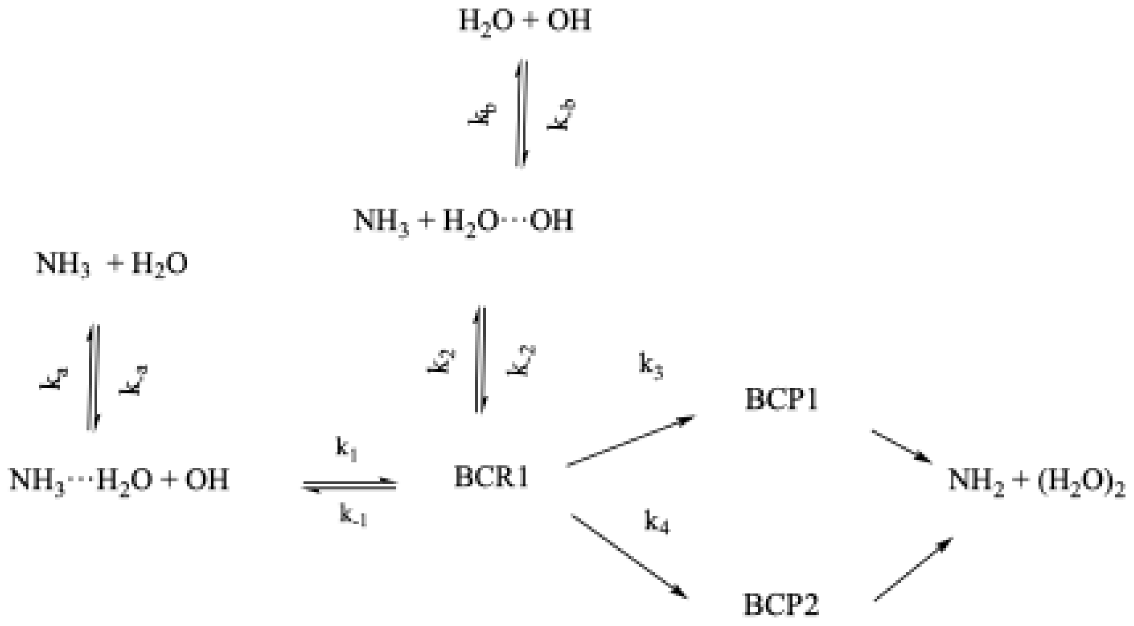

Figure S1: Kinetic scheme for the reaction of $NH_3\cdots H_2O$ + OH according the potential energy surface of Figure 2 in the main text. See Tables S13 and S16 below.

Table S3: Rate constants, in $cm^3\cdot molecule^{-1}\cdot s^{-1}$, or the reaction of $NH_3\cdots H_2O$ + OH at different relative humidity (RH) and at different temperatures T (in K)[a]

T	20%RH	40%RH	60%RH	80%RH	100%RH
250.00	2.52e-18	5.04e-18	7.56e-18	1.01e-17	1.26e-17
260.00	4.38e-18	8.76e-18	1.31e-17	1.75e-17	2.19e-17
270.00	7.46e-18	1.49e-17	2.24e-17	2.98e-17	3.73e-17
275.00	9.64e-18	1.93e-17	2.89e-17	3.85e-17	4.81e-17
280.00	1.24e-17	2.48e-17	3.71e-17	4.95e-17	6.18e-17
290.00	2.00e-17	3.99e-17	5.99e-17	7.98e-17	9.97e-17
298.00	2.88e-17	5.76e-17	8.64e-17	1.15e-16	1.44e-16
300.00	3.14e-17	6.28e-17	9.41e-17	1.25e-16	1.57e-16
310.00	4.81e-17	9.61e-17	1.44e-16	1.92e-16	2.39e-16
320.00	7.19e-17	1.44e-16	2.15e-16	2.86e-16	3.57e-16

a) Calculated by numerical integration according the scheme of Figure S1.

Table S4: Total rate constants, in $cm^3 \cdot molecule^{-1} \cdot s^{-1}$, or the reaction of NH_3 + OH at different relative humidity (RH) and at different temperatures T (in K).[a, b]

T	0%RH	20%RH	40%RH	60%RH	80%RH	100%RH
250.00	6.50e-14	6.50e-14	6.50e-14	6.50e-14	6.50e-14	6.50e-14
260.00	7.51e-14	7.51e-14	7.51e-14	7.51e-14	7.51e-14	7.51e-14
270.00	8.59e-14	8.59e-14	8.59e-14	8.59e-14	8.59e-14	8.59e-14
275.00	9.21e-14	9.21e-14	9.21e-14	9.21e-14	9.21e-14	9.21e-14
280.00	9.83e-14	9.83e-14	9.83e-14	9.83e-14	9.83e-14	9.84e-14
290.00	1.12e-13	1.12e-13	1.12e-13	1.13e-13	1.13e-13	1.13e-13
298.00	1.24e-13	1.24e-13	1.24e-13	1.24e-13	1.24e-13	1.24e-13
300.00	1.27e-13	1.27e-13	1.27e-13	1.27e-13	1.27e-13	1.27e-13
310.00	1.44e-13	1.44e-13	1.44e-13	1.44e-13	1.44e-13	1.44e-13
320.00	1.61e-13	1.61e-13	1.61e-13	1.62e-13	1.62e-13	1.62e-13

a) The values at 0% RH correspond to the NH_3 + OH naked reaction.
b) The total values at each % RH correspond to the values at 0% RH plus the values at the corresponding % RH in Table S3.

c) **Reaction of $NH_3 \cdots HNO_3$ + OH**

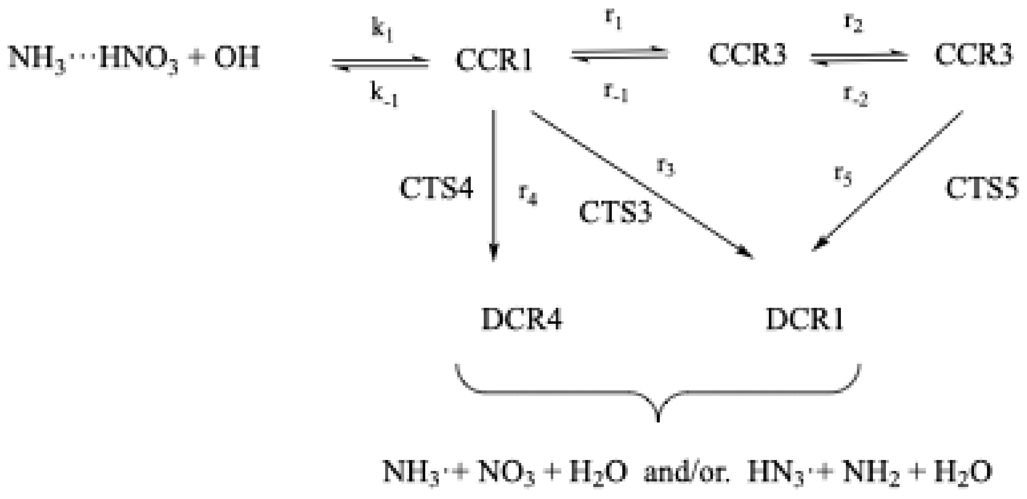

Figure S2: Kinetic scheme for the reaction of $NH_3\cdots HNO_3$ + OH according the potential energy surface of Figure 3 in the main text. See Tables S14 and S17 below.

Table S5. Calculated rate constants, k in $cm^3\cdot molecule^{-1}\cdot s^{-1}$, or the reaction of $NH_3\cdots HNO_3$ + OH at different temperatures (T in K).[a]

T	k
220.00	2.53e-16
230.00	3.67e-16
240.00	4.32e-16
250.00	4.91e-16
260.00	5.24e-16
270.00	5.13e-16
280.00	5.49e-16
290.00	5.81e-16
298.00	6.50e-16
300.00	6.64e-16
310.00	7.23e-16
320.00	7.63e-16

a) Calculated by numerical integration according the scheme of Figure S2.

d) **Reaction of NH_3·+ H_2O + NO_3 and reaction of HNO_3·+ H_2O + NH_2**

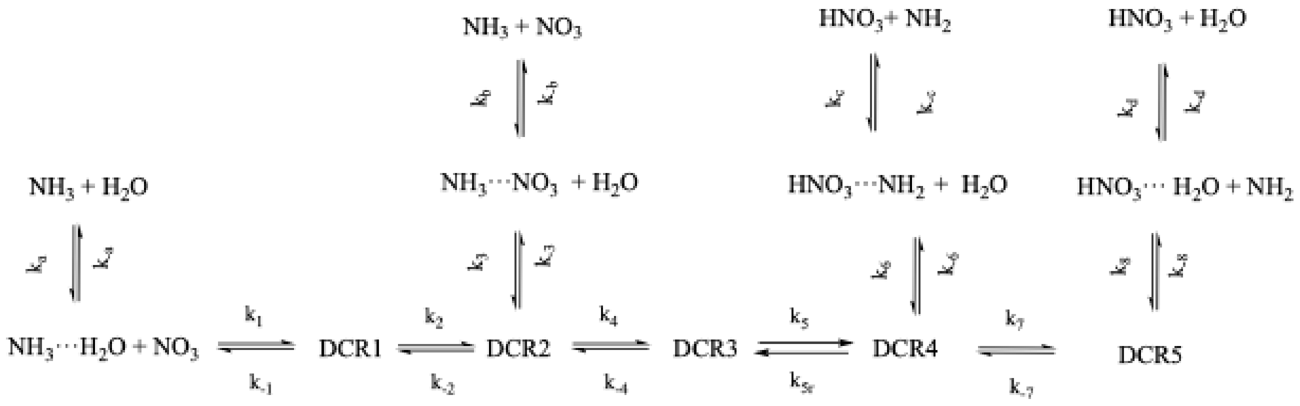

Figure S3: Kinetic scheme for the reaction of $NH_3 \cdots H_2O + NO_3$ and $HNO_3 \cdots H_2O + NH_2$ according the potential energy surface of Figure 5 in the main text. See Tables S15 and S18 below.

Table S6: Rate constants, in $cm^3 \cdot molecule^{-1} \cdot s^{-1}$, or the reaction of $HNO_3 \cdots H_2O + NH_2$ at different relative humidity (RH) and at different temperatures T (in K)[a]

T	20%RH	40%RH	60%RH	80%RH	100%RH
250.00	1.90e-15	3.75e-15	5.56e-15	7.32e-15	9.04e-15
260.00	2.35e-15	4.64e-15	6.85e-15	9.00e-15	1.11e-14
270.00	2.84e-15	5.58e-15	8.23e-15	1.08e-14	1.33e-14
275.00	3.06e-15	6.01e-15	8.85e-15	1.16e-14	1.42e-14
280.00	3.30e-15	6.46e-15	9.50e-15	1.24e-14	1.52e-14
290.00	3.85e-15	7.53e-15	1.10e-14	1.44e-14	1.76e-14
298.00	4.36e-15	8.49e-15	1.24e-14	1.62e-14	1.97e-14
300.00	4.43e-15	8.63e-15	1.26e-14	1.64e-14	2.00e-14
310.00	5.04e-15	9.78e-15	1.43e-14	1.85e-14	2.25e-14
325.00	7.41e-15	1.43e-14	2.08e-14	2.69e-14	3.26e-14

a) Calculated by numerical integration according the scheme of Figure S3

Table S7: Total rate constants, in $cm^3 \cdot molecule^{-1} \cdot s^{-1}$, or the reaction of $HNO_3 \cdots H_2O$ + NH_2 at different relative humidity (RH) and at different temperatures T (in K).[a, b]

T	0%RH	20%RH	40%RH	60%RH	80%RH	100%RH
250.00	1.91e-13	1.93e-13	1.95e-13	1.97e-13	1.99e-13	2.00e-13
260.00	1.87e-13	1.89e-13	1.92e-13	1.94e-13	1.96e-13	1.98e-13
270.00	1.84e-13	1.87e-13	1.90e-13	1.92e-13	1.95e-13	1.97e-13
275.00	1.83e-13	1.86e-13	1.89e-13	1.92e-13	1.94e-13	1.97e-13
280.00	1.81e-13	1.85e-13	1.88e-13	1.91e-13	1.94e-13	1.97e-13
290.00	1.80e-13	1.83e-13	1.87e-13	1.91e-13	1.94e-13	1.97e-13
298.00	1.79e-13	1.83e-13	1.87e-13	1.91e-13	1.95e-13	1.98e-13
300.00	1.78e-13	1.83e-13	1.87e-13	1.91e-13	1.95e-13	1.98e-13
310.00	1.78e-13	1.83e-13	1.87e-13	1.92e-13	1.96e-13	2.00e-13
325.00	1.77e-13	1.85e-13	1.92e-13	1.98e-13	2.04e-13	2.10e-13

a) The values at 0% RH correspond to the $HNO_3 + NH_2$ naked reaction from reference [1].
b) The total values at each % RH correspond to the values at 0% RH plus the values at the corresponding % RH in Table S6.

Table S8: Percentage of increment of the rate constant because of the relative humidity, relative to the naked reaction

T	20%RH	40%RH	60%RH	80%RH	100%RH
250.00	0.99	1.96	2.91	3.83	4.73
260.00	1.26	2.48	3.66	4.81	5.93
270.00	1.54	3.03	4.47	5.86	7.20
275.00	1.68	3.29	4.84	6.34	7.78
280.00	1.82	3.56	5.24	6.85	8.40
290.00	2.15	4.20	6.15	8.02	9.81
298.00	2.44	4.75	6.96	9.05	11.05
300.00	2.48	4.84	7.08	9.21	11.24
310.00	2.84	5.51	8.04	10.43	12.69
325.00	4.18	8.09	11.74	15.17	18.38

Regarding the naked $NO_3 + NH_3$ reaction, we have reported previous kinetic values at the supplementary material of reference [1], and in this work, we have recalculated by changing the data of the pre-reactive complex (reported at B3LYP in reference [1]), by values obtained at geometries optimized at QCISD. In Table S9 we have collected the relative energies and in Table S11 (column 0%RH) we have collected the rate constants, which are roughly two times greater than the values reported previously.

Table S9. Relative energies, relative energies plus Zero-Point Energies (ZPE), relative enthalpies at 298 K and relative free energies at 298K for the NO3 + NH3 -> HNO3 + NH2 reaction.

Stationary points	DE	D (E+ZPE)	DH(298K)	DG(298K
$NO_3 + NH_3$	0.00	0.00	0.00	0.00
CR	-3.99	-2.94	-2.72	3.82
TS	2.67	*3.61*	*2.18*	*12.81*
CP	-7.26	*-4.88*	*-5.35*	*2.68*
$HNO_3 + NH_2$	3.25	3.44	3.23	3.35

Table S10: Rate constants, in $cm^3 \cdot molecule^{-1} \cdot s^{-1}$, or the reaction of $NH_3 \cdots H_2O + NO_3$ at different relative humidity (RH) and at different temperatures T (in K)[a]

T	20%RH	40%RH	60%RH	80%RH	100%RH
250.00	1.49e-17	2.98e-17	4.48e-17	5.97e-17	7.46e-17
260.00	2.80e-17	5.60e-17	8.40e-17	1.12e-16	1.40e-16
270.00	5.01e-17	1.00e-16	1.50e-16	2.00e-16	2.50e-16
275.00	6.58e-17	1.32e-16	1.97e-16	2.63e-16	3.29e-16
280.00	8.57e-17	1.71e-16	2.57e-16	3.43e-16	4.28e-16
290.00	1.41e-16	2.82e-16	4.23e-16	5.64e-16	7.05e-16
298.00	2.05e-16	4.10e-16	6.15e-16	8.20e-16	1.02e-15
300.00	2.25e-16	4.49e-16	6.73e-16	8.98e-16	1.12e-15
310.00	3.46e-16	6.92e-16	1.04e-15	1.38e-15	1.73e-15
325.00	6.31e-16	1.26e-15	1.89e-15	2.52e-15	3.14e-15

a) Calculated by numerical integration according the scheme of Figure S3

Table S11: Total rate constants, in $cm^3 \cdot molecule^{-1} \cdot s^{-1}$, or the reaction of $NH_3 \cdots H_2O$ + NO_3 at different relative humidity (RH) and at different temperatures T (in K).[a, b]

T	0%RH	20%RH	40%RH	60%RH	80%RH	100%RH
250.00	6.02e-17	7.51e-17	9.00e-17	1.05e-16	1.20e-16	1.35e-16
260.00	7.27e-17	1.01e-16	1.29e-16	1.57e-16	1.85e-16	2.13e-16
270.00	8.71e-17	1.37e-16	1.87e-16	2.37e-16	2.87e-16	3.37e-16
275.00	9.50e-17	1.61e-16	2.27e-16	2.92e-16	3.58e-16	4.24e-16
280.00	1.03e-16	1.89e-16	2.74e-16	3.60e-16	4.46e-16	5.31e-16
290.00	1.20e-16	2.62e-16	4.03e-16	5.44e-16	6.85e-16	8.26e-16
298.00	1.36e-16	3.42e-16	5.47e-16	7.52e-16	9.57e-16	1.16e-15
300.00	1.41e-16	3.65e-16	5.90e-16	8.14e-16	1.04e-15	1.26e-15
310.00	1.62e-16	5.08e-16	8.54e-16	1.20e-15	1.55e-15	1.89e-15
325.00	1.99e-16	8.30e-16	1.46e-15	2.09e-15	2.72e-15	3.34e-15

a) The values at 0% RH correspond to the $NO_3 + NH_3$ naked reaction.
b) The total values at each % RH correspond to the values at 0% RH plus the values at the corresponding % RH in Table S10.

Table S12. Percentage of increment of the rate constant of the $NH_3 + NO3 + H_2O$ due to the relative humidity, relative to the naked reaction

T	20%RH	40%RH	60%RH	80%RH	100%RH
250.00	24.78	49.56	74.34	99.11	123.88
260.00	38.53	77.05	115.56	154.07	192.57
270.00	57.45	114.88	172.30	229.70	287.09
275.00	69.25	138.47	207.68	276.86	346.02
280.00	83.08	166.12	249.13	332.12	415.07
290.00	117.31	234.57	351.76	468.89	585.96
298.00	150.53	300.95	451.27	601.50	751.62
300.00	159.80	319.49	479.07	638.53	797.88

310.00	213.41	426.61	639.61	852.41	1065.01
325.00	317.01	633.57	949.70	1265.38	1580.63

Table S13: Kinetic equations involved for the determination of the whole rate constants by numerical integration according to Scheme displayed in Figure S1 for the reaction of $NH_3\cdots H_2O$ + OH.

d[nh3] $= - k_a$[nh3][h2o] + $k_{\_a}$[nh3-h2o]

d[h2o] $= - k_a$[nh3][h2o] + $k_{\_a}$[nh3-h2o] - k_b[h2o][oh] + $k_{\_b}$[h2o-oh]

d[oh] $= - k_1$[nh3-h2o][oh] + $k_{\_1}$[bcr1] – k_b[h2o][oh] + $k_{\_b}$[h2o-oh]

d[nh3-h2o] $= - k_1$[nh3-h2o][oh] + $k_{\_1}$[bcr1] + k_a[nh3][h2o] - $k_{\_a}$[nh3-h2o]

d[h2o-oh] = - $k_{\_b}$[h2o-oh] + k_b[h2o][oh] + $k_{\_2}$[bcr1] – k_2[nh3][h2o-oh]

d[bcr1] = k_1[nh3-h2o][oh] - k_{-1}[bcr1] - $k_{\_2}$[bcr1] + k_2[nh3][h2o-oh] – k_3[bcr1] – k_4[bcr1]

d[bcp1] = + k_3[bcr1]

d[bcp2] = + k_4[bcr1]

Table S14: Kinetic equations involved for the determination of the whole rate constants by numerical integration according to Scheme displayed in Figure S2 for the reactions of $NH_3\cdots HNO_3$ + OH

d[hno3nh3]. = - k_1[hno3nh3][oh] + k_{-1}[ccr1]

d[oh] = - k_1[hno3nh3][oh] + k_{-1}[ccr1]

d[ccr1] = k_1[hno3nh3][oh] - k_{-1}[ccr1] -r_1[ccr2] + r_{-1}[ccr2] – r_3[ccr1] – r_4[ccr1]

d[ccr2] =. r_1[ccr1] – r_{-1}[ccr2] – r_2[ccr2] + r_{-2}[ccr3]

d[ccr3]. = r2[ccr2] – r-2[ccr3] – r5[ccr3]

d(dcr4]. = r_4[ccr1]

d[dcr1]. = r_3[ccr1] + r_5[ccr3]

Table S15: Kinetic equations involved for the determination of the whole rate constants by numerical integration according to Scheme displayed in Figure S3.

(a) Reaction $NH_3\cdots H_2O + NO_3$

$d[nh3] \quad = -k_a[nh3][h2o] + k_{\_a}[nh3][[h2o] - k_b[nh3][no3]+k_{\_b}[nh3no3]$

$d[h2o] \quad = -k_a[nh3][h2o] + k_{\_a}[nh3][h2o] - k_3[nh3no3][h2o] + k\_3[dcr2]$

$d[no3] \quad = -k_1[nh3h2o][no3] + k_{\_1}[dcr1] -k_b[nh3][no3] + k_{\_b}[nh3no3]$

$d[nh3h2o] \ = -k_1[nh3h2o][no3] + k_{\_1}[dcr1] + k_a[nh3][h2o] - k_{\_a}[nh3h2o]$

$d[nh3no3] \ = -k_3[nh3no3[h2o] + k_{\_3}[dcr2] + k_b[nh3][no3] - k_{\_b}[nh3no3]$

$d[dcr1] \quad = \ k_1[nh3h2o][no3] - k_{\_1}[dcr1] - k_{\_2}[dcr1] + k_2[dcr2]$

$d[dcr2] \quad = \ k_2[dcr1] - k_{\_2}[dcr2] -k_4[dcr2] + k_{\_4}[dcr3] + k_3[nh3no3][h2] - k_{\_3}[dcr2]$

$d[dcr3] \quad = \ k_4[dcr2] - k_{\_4}[dcr3] - k_5[dcr3]$

$d[dcr4] \quad = \ k_5[dcr3]$

(b) Reaction $HNO_3\cdots H_2O + NH_2$

$d[hno3] \quad = -k_d[hno3][h2o] + k_{\_d}[hno3h2o] - k_c[hno3][nh2] + k_{\_c}[hno3nh2]$

$d[h2o] \quad = -k_d[hno3][h2o] + k_{\_d}[hno3h2o] - k_6[hno3nh2][h2o] + k_{\_6}[dcr4]$

$d[nh2] \quad = -k_c[hno3][nh2] + k_{\_c}[hno3nh2] - k_8[hno3h2o][nh2] + k_{\_8}[dcr5$

$d[hno3h2o] = k_d[hno3][h2o] - k_{\_d}[hno3h2o] - k8[hno3h2o][nh2] + k_{\_8}[dcr5]$

$d[hno3nh2] = k_c[hno3][nh2] - k_{\_c}[hno3nh2] - k_6[hno3nh2][h2o] + k_{\_6}[dcr4]$

$d[dcr5] \quad = k_8[hno3nh2][nh2] - k_{\_8}[dcr5] + k_7[dcr4] - k_{\_7}[dcr5]$

$d[dcr4] \quad = k_6[hno3nh2][h2o] - k_{\_6}[dcr4] - k_7[dcr4] + k_{\_7}[dcr5] - k_{5r}[dcr4]$

$d[dcr3] \quad = k_{5r}[dcr4]$

Table S16: Rate constants of the elementary reactions (in s^{-1}) at different temperatures (T in K), for the process described in Figure S1 and Table S13 for the reaction of $NH_3\cdots H_2O$ + OH.

T	k_a	k_{-a}	K_b	k_{-b}	k_1	k_{-1}	k_2	k_{-2}	k_3	Tunneling(k_3)	k_4	Tunneling (k_4)
250	1.07E-06	1.08E+14	3.57E-07	3.13E+13	1.07E-06	2.08E+12	1.07E-06	7.21E+12	3.92E+04	10.54	1.45E+05	12.61
260	1.16E-06	1.68E+14	3.87E-07	4.55E+13	1.16E-06	3.7E+12	1.16E-06	1.37E+13	7.84E+04	8.83	2.64E+05	9.78
270	1.25E-06	2.54E+14	4.17E-07	6.42E+13	1.25E-06	6.32E+12	1.25E-06	2.5E+13	1.50E+05	7.54	4.74E+05	7.95
275	1.30E-06	3.07E+14	4.32E-07	7.56E+13	1.30E-06	8.14E+12	1.30E-06	3.31E+13	2.04E+05	7.01	6.29E+05	7.26
280	1.35E-06	3.71E+14	4.48E-07	8.86E+13	1.35E-06	1.04E+13	1.35E-06	4.34E+13	2.75E+05	6.53	8.31E+05	6.70
290	1.44E-06	5.31E+14	4.81E-07	1.2E+14	1.44E-06	1.65E+13	1.45E-06	7.29E+13	4.85E+05	5.74	1.42E+06	5.75
298	1.52E-06	6.94E+14	5.08E-07	1.5E+14	1.53E-06	2.33E+13	1.53E-06	1.08E+14	7.45E+05	5.21	2.15E+06	5.21
300	1.54E-06	7.4E+14	5.14E-07	1.58E+14	1.55E-06	2.54E+13	1.55E-06	1.18E+14	8.26E+05	5.10	2.37E+06	5.06
310	1.65E-06	1.01E+15	5.49E-07	2.06E+14	1.65E-06	3.79E+13	1.65E-06	1.86E+14	1.36E+06	4.56	3.85E+06	4.52
325	1.76E-06	1.36E+15	5.85E-07	2.64E+14	1.76E-06	5.53E+13	1.76E-06	2.85E+14	2.19E+06	4.16	6.10E+06	4.07

Table S17: Rate constants of the elementary reactions (in s^{-1}) at different temperatures (T in K), of the process described in Figure S2 and Table S14 for the reactions of $NH_3\cdots HNO_3$ + OH.

T	k_1	k_{-1}	r_1	r_{-1}	r_2	r_{-2}	r_3	Tunneling (r_3)	r_4	Tunneling (r_4)	r_5	Tunneling (r_5)
220	4.16E-07	6.58E+13	1.68E+07	1.97E+12	5.57E+12	1.36E+09	2.00E+00	1.98	1.17E+05	3534.74	6.22E+09	294786.73
230	4.55E-07	1.13E+14	3.05E+07	2.14E+12	5.76E+12	1.96E+09	5.68E+00	1.86	1.42E+05	1486.91	6.45E+09	146258.50
240	4.95E-07	1.87E+14	5.31E+07	2.32E+12	5.94E+12	2.74E+09	1.48E+01	1.76	1.75E+05	694.44	6.69E+09	77430.56
250	5.38E-07	2.97E+14	8.84E+07	2.49E+12	6.13E+12	3.75E+09	3.58E+01	1.68	2.17E+05	352.85	6.93E+09	43312.50
260	5.81E-07	4.55E+14	1.42E+08	2.67E+12	6.30E+12	5.01E+09	8.08E+01	1.61	2.73E+05	195.00	7.18E+09	25371.02
270	6.26E-07	6.76E+14	2.20E+08	2.85E+12	6.47E+12	6.56E+09	1.72E+02	1.56	3.46E+05	115.33	7.44E+09	15500.00
280	6.74E-07	9.77E+14	3.31E+08	3.03E+12	6.64E+12	8.45E+09	3.47E+02	1.51	4.43E+05	72.86	7.70E+09	9833.97
290	7.24E-07	1.38E+15	4.85E+08	3.21E+12	6.79E+12	1.07E+10	6.65E+02	1.46	5.70E+05	48.72	7.97E+09	6479.67
298	7.64E-07	1.79E+15	6.46E+08	3.36E+12	6.92E+12	1.28E+10	1.09E+03	1.44	7.00E+05	36.46	8.19E+09	4706.90
300	7.74E-07	1.90E+15	6.93E+08	3.39E+12	6.95E+12	1.33E+10	1.22E+03	1.42	7.37E+05	33.96	8.24E+09	4359.79
310	8.26E-07	2.57E+15	9.69E+08	3.58E+12	7.10E+12	1.64E+10	2.16E+03	1.39	9.55E+05	24.81	8.52E+09	3032.03
320	8.80E-07	3.41E+15	1.33E+09	3.76E+12	7.25E+12	2.00E+10	3.69E+03	1.37	1.24E+06	18.82	8.81E+09	2164.62

Table S18: Rate constants of the elementary reactions (in s^{-1}) at different temperatures (T in K), of the process described in Figure S3 and Table S15 for the $NH_3\cdots H_2O + NO_3$ and $HNO_3\cdots H_2O + NH_2$ reactions.

T	k_{-7}	k_7	k_{-6}	k_6	k_{-8}	k_8	k-c	k_c	k_{-d}	k_d	k_{5r}	Tunneling (k_{5r})	k_5	Tunneling (k_5)
250	3.61E+07	3.83E+09	4.85E+13	1.43E-06	1.00E+12	1.43E-06	5.18E+10	7.16E-07	3.11E+11	7.15E-07	8.67E+05	2.40	1.37E+12	2.40
260	5.63E+07	5.37E+09	8.00E+13	1.55E-06	1.71E+12	1.55E-06	1.05E+11	7.75E-07	6.28E+11	7.75E-07	1.36E+06	2.28	1.39E+12	2.27
270	8.52E+07	7.37E+09	1.27E+14	1.67E-06	2.81E+12	1.67E-06	2.04E+11	8.36E-07	1.2E+12	8.35E-07	2.06E+06	2.17	1.42E+12	2.17
275	1.04E+08	8.56E+09	1.58E+14	1.73E-06	3.55E+12	1.73E-06	3.51E+11	1.08E-06	1.64E+12	8.66E-07	2.50E+06	2.12	1.43E+12	2.12
280	1.25E+08	9.89E+09	1.95E+14	1.79E-06	4.46E+12	1.79E-06	5.91E+11	1.39E-06	2.2E+12	8.98E-07	3.02E+06	2.07	1.44E+12	2.08
290	1.80E+08	1.30E+10	2.91E+14	1.93E-06	6.86E+12	1.92E-06	9.41E+11	1.35E-06	3.87E+12	9.64E-07	4.31E+06	1.99	1.46E+12	2.00
298	2.36E+08	1.60E+10	3.93E+14	2.04E-06	9.49E+12	2.03E-06	1.11E+12	1.11E-06	5.9E+12	1.02E-06	5.64E+06	1.93	1.47E+12	1.93
300	2.52E+08	1.69E+10	4.21E+14	2.06E-06	1.03E+13	2.06E-06	1.72E+12	1.55E-06	6.53E+12	1.03E-06	6.02E+06	1.92	1.47E+12	1.91
310	3.46E+08	2.15E+10	5.98E+14	2.20E-06	1.50E+13	2.20E-06	2.79E+12	1.64E-06	1.07E+13	1.10E-06	8.21E+06	1.85	1.49E+12	1.85
325	5.38E+08	3.01E+10	9.68E+14	2.42E-06	2.52E+13	2.41E-06	7.24E+10	2.96E-08	2.11E+13	1.21E-06	1.26E+07	1.76	1.52E+12	1.77

T	k_a	k_{-a}	k_1	k_{-1}	k_2	k_{-2}	k_3	k_{-3}	k_b	k_{-b}	k_4	k_{-4}
250	1.07E-06	1.08E+14	3.22E-06	4.99E+14	1.65E+12	7.26E+12	3.22E-06	2.31E+16	1.07E-06	2.07E+14	3.75E+09	1.53E+10
260	1.16E-06	1.68E+14	3.48E-06	6.49E+14	1.85E+12	7.56E+12	3.49E-06	2.99E+16	1.16E-06	2.68E+14	4.49E+09	1.74E+10

270	1.25E-06	2.54E+14	3.75E-06	8.27E+14	2.05E+12	7.83E+12	3.76E-06	3.78E+16	1.25E-06	3.40E+14	5.30E+09	1.96E+10
275	1.30E-06	3.07E+14	3.90E-06	9.27E+14	2.17E+12	7.97E+12	3.90E-06	4.23E+16	1.30E-06	3.80E+14	5.73E+09	2.07E+10
280	1.35E-06	3.71E+14	4.04E-06	1.04E+15	2.28E+12	8.11E+12	4.04E-06	4.72E+16	1.34E-06	4.23E+14	6.19E+09	2.18E+10
290	1.44E-06	5.31E+14	4.33E-06	1.27E+15	2.50E+12	8.38E+12	4.34E-06	5.79E+16	1.44E-06	5.18E+14	7.14E+09	2.41E+10
298	1.52E-06	6.94E+14	4.58E-06	1.48E+15	2.69E+12	8.58E+12	4.58E-06	6.75E+16	1.52E-06	6.04E+14	7.95E+09	2.6E+10
300	1.54E-06	7.4E+14	4.64E-06	1.54E+15	2.74E+12	8.64E+12	4.64E-06	7.00E+16	1.54E-06	6.27E+14	8.16E+09	2.64E+10
310	1.65E-06	1.01E+15	4.95E-06	1.84E+15	2.98E+12	8.89E+12	4.95E-06	8.36E+16	1.65E-06	7.47E+14	9.23E+09	2.88E+10
325	1.81E-06	1.57E+15	5.44E-06	2.34E+15	3.36E+12	9.28E+12	5.45E-06	1.07E+17	1.81E-06	9.52E+14	1.10E+10	3.25E+10

Table S19. Cartesian Coordinates (in Angstrom) of the stationary points investigated in this work

H2O QCISD/6-311+G(2df,2p)

H	0.000000	0.756586	-0.468730
O	0.000000	0.000000	0.117182
H	0.000000	-0.756586	-0.468730

NH2 QCISD/6-311+G(2df,2p)

N	0.000000	0.000000	0.141274
H	0.000000	0.802427	-0.494460
H	0.000000	-0.802427	-0.494460

NH3 QCISD/6-311+G(2df,2p)

N	0.000000	0.112590	0.000000
H	0.469269	-0.262703	0.812809
H	-0.938538	-0.262726	0.000000
H	0.469269	-0.262703	-0.812809

OH QCISD/6-311+G(2df,2p)

O	0.000000	0.000000	0.107651
H	0.000000	0.000000	-0.861206

H2O···OH QCISD/6-311+G(2df,2p)

O	-0.033629	-1.279538	0.000000
H	-0.055797	0.662533	0.000000
H	0.296932	-1.756492	0.761361
H	0.296932	-1.756492	-0.761361
O	-0.033629	1.635844	0.000000

H2O···H2O QCISD/6-311+G(2df,2p)

H	0.071582	0.575312	0.000000
O	-0.001046	1.534599	0.000000
H	0.902565	1.846874	0.000000
O	-0.001046	-1.402895	0.000000
H	-0.478709	-1.737910	0.759532
H	-0.478709	-1.737910	-0.759532

NH3···H2O QCISD/6-311+G(2df,2p)

N	1.401235	0.021640	0.000271
H	1.773139	-0.048204	0.938232
H	1.639989	-0.837968	-0.476811
H	1.903822	0.766198	-0.464134
H	-0.613689	0.028051	0.001152
O	-1.571290	-0.104527	-0.000210
H	-1.941587	0.776659	0.001343

NH2..H2O QCISD/6-311+G(2df,2p)

H	0.866788	-1.838147	0.000000

O	-0.038565	-1.531201	0.000000
H	0.028469	-0.570398	0.000000
N	-0.038565	1.491962	0.000000
H	-0.158393	2.107209	0.807555
H	-0.158393	2.17209	-0.807555

NO3 QCISD/6-311+G(2df,2p)

N	0.000000	0.000000	0.000000
O	0.000000	1.226095	0.000000
O	1.061830	-0.613048	0.000000
O	-1.061830	-0.613048	0.000000

ACR1 QCISD/6-311+G(2df,2p)

N	0.000233	-1.285468	0.000000
H	-0.471114	-1.661651	0.812286
H	-0.471114	-1.661651	-0.812286
H	0.937242	-1.666731	0.000000
H	0.001498	0.683080	0.000000
O	0.000233	1.663154	0.000000

ACR2 QCISD/6-311+G(2df,2p)

N	-0.076923	-1.435106	0.000000
H	-0.331319	-0.893319	0.814249
H	0.926858	-1.550579	0.000000
H	-0.331319	-0.893319	-0.814249
O	-0.076923	1.479440	0.000000
H	0.889632	1.547438	0.000000

ATS1 QCISD/6-311+G(2df,2p)

N	1.151134	-0.044897	-0.102189
H	1.304792	-0.541131	0.770983
H	1.339869	0.936446	0.083206
H	0.030518	-0.156129	-0.349752
O	-1.179788	-0.092251	-0.011196
H	-1.294807	0.813101	0.300454

ACP1 QCISD/6-311+G(2df,2p) (2A')

H	0.866788	-1.838147	0.000000
O	-0.038565	-1.531201	0.000000
H	0.028469	-0.570398	0.000000
N	-0.038565	1.491962	0.000000
H	-0.158393	2.107209	0.807555
H	-0.158393	2.107209	-0.807555

BCR1 QCISD/6-311+G(2df,2p)

N	-1.695578	-0.410371	0.011089
H	-2.279412	-0.622313	0.809047
H	-2.278092	-0.493145	-0.811517
H	-0.989068	-1.135642	-0.045930
O	1.523204	-1.176609	0.012742
H	1.371262	-0.210368	-0.008198
O	0.472198	1.440817	-0.080844
H	0.485391	2.191180	0.511157
H	-0.404252	1.029219	0.012636

BTS1 QCISD/6-311+G(2df,2p)

N	1.477825	-0.927219	-0.104987
H	0.569087	-1.360564	0.041874
H	1.944299	-0.940186	0.797958
H	1.262431	0.190417	-0.329997
O	0.719446	1.265140	0.011121
H	-0.192598	0.969814	0.142291
O	-1.854603	-0.260673	0.021792
H	-2.573385	-0.237946	0.654262
H	-2.273356	-0.166743	-0.834783

BCP1 QCISD/6-311+G(2df,2p)

N	-0.937292	-2.580637	0.000000
H	-1.025074	-3.202195	0.807103
H	-1.025074	-3.202195	-0.807103
H	-0.870842	-0.476997	0.000000
O	-0.914000	0.483436	0.000000
H	0.000000	0.776976	0.000000
O	1.703005	1.915410	0.000000
H	1.585000	2.489050	-0.758010
H	1.585000	2.489050	0.758010

BTS2 QCISD/6-311+G(2df,2p)

N	0.318084	0.793206	-0.047069
H	0.618716	1.393489	-0.810221
H	0.463518	1.319571	0.810876

H	1.060462	-0.108521	-0.037735
O	2.189966	-0.605278	-0.060782
H	2.649151	-0.191603	0.680083
H	-1.611800	-0.014443	-0.042868
O	-2.541258	-0.243926	0.054902
H	-2.596303	-1.157303	-0.223609

BCP2 QCISD/6-311+G(2df,2p)

N	-1.658388	-0.644690	-0.002270
H	-2.483235	-1.245725	-0.012872
H	-0.851728	-1.276254	-0.008004
H	2.141604	-1.143287	-0.443846
O	1.357379	-0.991224	0.081119
H	1.194821	-0.040457	0.029315
H	-0.662365	1.097638	-0.046136
O	0.163506	1.598840	-0.093317
H	0.102540	2.259984	0.595020

NH3-HNO3 QCISD/6-311G(d,p)

N	0.000000	0.903223	0.000000
O	-1.013694	-0.010627	0.000000
H	-0.567025	-0.905917	0.000000
O	-0.351614	2.049317	0.000000
O	1.136589	0.468308	0.000000
N	0.174642	-2.453573	0.000000
H	1.165340	-2.225702	0.000000
H	0.004472	-3.035955	0.814771

H 0.004472 -3.035955 -0.814771

NH3-HNO3 QCISD/6-311+G(2df,2p)

N 0.000000 0.905979 0.000000

O -1.003698 -0.008589 0.000000

H -0.555061 -0.906024 0.000000

O -0.353322 2.048853 0.000000

O 1.135665 0.474420 0.000000

N 0.169223 -2.463752 0.000000

H 1.154831 -2.231323 0.000000

H -0.006743 -3.037856 0.814228

H -0.006743 -3.037856 -0.814228

CCR1 QCISD/6-311+G(2df,2p)

N -1.282379 -0.164152 0.027995

O -0.935581 1.112514 -0.221265

H 0.067690 1.190214 -0.074309

O -2.443719 -0.417430 -0.083944

O -0.388338 -0.933512 0.345837

N 1.683622 1.528889 0.108932

H 1.898834 1.883419 1.032261

H 2.217267 0.674499 -0.016267

H 2.034528 2.207564 -0.554883

O 2.452751 -1.506162 -0.202085

H 1.492071 -1.552137 -0.053638

CTS1 QCISD/6-311+G(2df,2p)

N	0.000382	-0.093627	-0.040370
O	0.067486	-0.628722	1.047647
O	0.644270	-0.322125	-1.018886
O	-0.925167	0.897963	-0.182652
H	-1.389465	0.930981	0.679196
N	-3.270216	-0.126967	1.441235
H	-3.207908	-1.113735	1.655986
H	-3.737269	-0.045420	0.547032
H	-3.872174	0.296194	2.134952
O	-1.525384	1.342922	2.657411
H	-1.033324	0.506917	2.705603

CCR2 QCISD/6-311+G(2df,2p)

N	-0.084954	-0.219393	-0.382147
O	-1.283557	-0.295874	-0.569654
O	0.794722	-0.622562	-1.081768
O	0.323467	0.394548	0.757864
H	-0.495192	0.702593	1.223609
N	-3.343760	-0.082589	1.732345
H	-2.933273	-0.285611	0.831482
H	-4.119126	0.551415	1.608408
H	-3.676081	-0.938922	2.153095
O	-1.665881	1.428066	2.243430
H	-1.764365	1.013328	3.110336

CTS2 QCISD/6-311+G(2df,2p)

N	-0.071411	-0.173978	-0.410818

O	-1.273128	-0.104732	-0.572280
O	0.752322	-0.588906	-1.168481
O	0.415774	0.272094	0.778667
H	-0.362915	0.601174	1.286962
N	-3.423285	-0.035471	1.723435
H	-2.992044	-0.137643	0.814236
H	-4.152096	0.660304	1.649034
H	-3.852402	-0.916067	1.973207
O	-1.492865	1.321204	2.418502
H	-1.796717	0.746403	3.134690

CCR3 QCISD/6-311+G(2df,2p)

N	0.161203	-0.032115	-0.159065
O	-0.173849	-0.646105	1.006611
O	1.169498	-0.422557	-0.665244
O	-0.590437	0.836114	-0.555873
H	-2.830880	1.099354	1.385004
H	-1.017643	-0.222067	1.299291
O	-2.491429	0.383470	1.990138
N	-3.266853	2.349663	0.168264
H	-2.503837	2.256214	-0.491152
H	-4.127683	2.203866	-0.342725
H	-3.273430	3.309144	0.488854

CTS3 QCISD/6-311+G(2df,2p)

N	-1.117982	-0.134124	0.014950
O	-2.314963	0.015118	0.094550

O	-0.369626	0.727359	0.653869
O	-0.568683	-1.055708	-0.593680
H	1.230635	-1.332819	0.042660
N	2.084506	-0.835263	0.305946
H	2.206417	-0.892476	1.308227
H	2.887466	-1.241178	-0.155173
H	1.778986	0.295618	-0.074758
O	1.242307	1.301120	-0.578695
H	1.218552	2.053452	0.024427

CTS4 QCISD/6-311+G(2df,2p)

N	-1.317777	-0.104842	0.047644
O	-0.866270	1.097584	-0.400282
H	0.096594	1.120767	-0.163556
O	-2.473779	-0.318816	-0.151922
O	-0.505713	-0.823219	0.600753
N	1.818013	1.086921	0.291853
H	1.930646	0.872320	1.279811
H	1.997208	0.048608	-0.267953
H	2.641700	1.621191	0.022949
O	2.371089	-1.081064	-0.451773
H	1.629588	-1.533320	-0.021942

CTS5 QCISD/6-311+G(2df,2p)

N	1.200006	0.155112	-0.009461
O	1.012518	-0.880541	0.730376
O	2.172152	0.843418	0.112657

O	0.297886	0.409261	-0.865894
H	-1.649009	-0.601153	-0.110028
H	-0.103486	-1.266259	0.225920
O	-1.018258	-1.322224	-0.433913
N	-2.587496	0.739668	0.340458
H	-2.081086	1.500927	-0.096098
H	-3.534876	0.763047	-0.013762
H	-2.633502	0.940660	1.331183

NO3···NH3 QCISD/6-311+G(2df,2p)

N	-0.736926	0.000057	0.000000
O	-0.743760	-0.612428	1.060902
O	-0.743760	-0.612428	-1.060902
O	-0.743760	1.225082	0.000000
N	2.186827	-0.000195	0.000000
H	2.567086	0.468425	0.811822
H	2.567086	0.468425	-0.811822
H	2.566770	-0.937692	0.000000

HNO3···NH2 QCISD/6-311+G(2df,2p)

O	-2.023162	-0.177139	-0.000370
N	-0.855723	0.073169	-0.000047
O	-0.316161	1.157826	-0.000080
O	-0.035663	-1.015730	0.000261
H	0.881869	-0.646362	0.000389

N	2.562131	-0.024572	0.000245
H	2.664189	0.991333	-0.000454
H	3.508975	-0.404808	0.000196

HNO3···H2O QCISD/6-311+G(2df,2p)

N	0.866219	0.069109	-0.003887
O	0.269778	1.127015	-0.014227
O	2.044328	-0.117066	0.006385
O	0.104400	-1.063461	-0.002661
H	-0.824305	-0.738745	-0.011623
O	-2.408375	-0.020259	-0.068151
H	-2.147502	0.899804	0.018616
H	-3.103689	-0.160922	0.574881

DCR1 QCISD/6-311+G(2df,2p) i2-1-b3l-qci-b2xxx-f

N	0.088265	0.082695	-0.031336
O	0.088328	0.066113	1.196164
O	1.132680	0.129809	-0.659897
O	-0.992233	0.022676	-0.611493
N	2.818609	3.016333	-1.036097
H	2.857223	2.014576	-1.176795
H	3.595650	3.262435	-0.437494
H	2.985703	3.450059	-1.934347
O	0.050662	2.856509	-0.142757
H	0.962738	3.078072	-0.383887
H	-0.218449	3.524956	0.485158

DCR1 B3LYP/6-311+G(2df,2p)

N	-1.384750	-0.249412	-0.529610
O	-1.664158	0.878440	-0.950146
O	-2.203191	-0.781996	0.230854
O	-0.365246	-0.826085	-0.866926
N	2.739986	0.000092	0.724546
H	3.411126	0.671245	0.371285
H	2.391663	-0.529938	-0.066198
H	3.236805	-0.642909	1.329222
O	0.122993	0.781642	1.682270
H	1.059737	0.623085	1.451161
H	0.099385	1.618552	2.150614

DTS1 B3LYP/6-311+G(2df,2p)

N	0.007157	-0.336510	-0.157639
O	-0.355906	0.447909	0.709759
O	-0.077811	-1.554701	0.013788
O	0.451581	0.072146	-1.233332
N	2.840156	-0.371451	1.013580
H	3.447723	0.040512	0.315153
H	3.037009	-1.365151	1.031203
H	3.118987	0.007041	1.912595
O	1.703279	1.553858	3.067362
H	1.280257	1.143833	2.304399
H	0.989567	1.921514	3.594132

DCR2 QCISD/6-311+G(2df,2p)

N	0.027743	0.050327	-0.016630
O	0.148661	-0.001469	1.196569
O	1.010324	0.034082	-0.751935
O	-1.082065	0.092858	-0.538622
N	0.164081	2.962851	0.074497
H	-0.729518	3.434538	0.124826
H	0.640190	3.334295	-0.737111
H	0.693251	3.252638	0.888443
O	1.288479	2.419924	2.882712
H	0.991266	1.604013	2.475500
H	1.949066	2.153286	3.521101

DCR2 B3LYP/6-311+G(2df,2p)

N	-0.785612	-0.304417	-0.776140
O	-0.947277	0.745355	-0.170692
O	-0.958936	-1.399608	-0.233190
O	-0.462646	-0.309530	-1.970106
N	2.107367	-0.375171	0.224676
H	2.809667	0.095917	-0.331481
H	2.439293	-1.311865	0.416130
H	2.013077	0.120228	1.105887
O	0.788050	1.457714	2.349133
H	0.077609	1.345151	1.707957
H	0.362124	1.480529	3.209614

DTS2 QCISD/6-311+G(2df,2p)

O	0.165082	0.969339	0.045391

N	0.172753	-0.241721	0.028224
O	0.413473	-0.802029	-1.096597
O	-0.047424	-0.973722	0.967783
H	3.480556	-1.046069	0.996553
N	2.988552	-0.184386	0.799676
H	2.793412	0.257774	1.690883
H	3.641113	0.412234	0.308217
H	0.855760	1.702627	2.517737
O	1.272329	1.119813	3.154331
H	0.705393	0.347139	3.158802

DTS2 B3LYP/6-311+G(2df,2p)

O	-0.627776	0.451440	0.784027
N	-0.011515	-0.253934	-0.029826
O	0.827025	0.330993	-0.752555
O	-0.195021	-1.449616	-0.143873
H	3.535375	-1.363781	1.408140
N	3.031635	-0.601777	0.979375
H	2.735363	0.090643	1.660039
H	3.567018	-0.183659	0.233624
H	0.703524	1.389864	2.198922
O	1.546655	1.500893	2.654880
H	1.328716	1.649933	3.578250

DCR3 QCISD/6-311+G(d,p)

O	-1.949858	-1.052445	0.058648
N	-1.165493	-0.133290	0.020494

O	-0.090146	-0.226131	0.770101
O	-1.311288	0.884188	-0.675341
H	0.299023	1.653245	-0.356232
N	1.165475	1.486753	0.166539
H	1.837353	0.785686	-0.156388
H	1.234843	1.832257	1.113025
H	1.573159	-1.239915	0.107716
O	2.417661	-0.975976	-0.275591
H	2.524796	-1.542606	-1.039888

DCR3 B3LYP/6-311+G(2df,2p)

O	-1.005330	-0.089174	-0.324102
N	0.108608	-0.451892	-0.000628
O	0.975077	0.516448	0.235145
O	0.476832	-1.617809	0.118334
H	2.353869	-1.259941	0.824976
N	2.899142	-0.428174	1.005983
H	2.759623	0.106429	1.857067
H	3.475481	-0.054161	0.273123
H	1.151958	1.559709	2.122529
O	1.836735	1.537479	2.804171
H	1.403742	1.733882	3.638434

DTS3 QCISD/6-311+G(2df,2p)

O	-2.112672	-0.899610	-0.070468
N	-1.165157	-0.168911	0.039600
O	-0.151845	-0.473191	0.733813

O	-1.143550	0.972250	-0.554500
H	0.105481	1.418398	-0.240763
N	1.167013	1.520721	0.160191
H	1.892866	0.877743	-0.161800
H	1.352549	1.957374	1.054567
H	1.577475	-1.259357	0.032998
O	2.442051	-0.952574	-0.264040
H	2.786759	-1.631833	-0.841972

DTS3 QCISD/6-311+G(d,p)

O	-2.082240	-0.914431	-0.056898
N	-1.153246	-0.154309	0.039150
O	-0.144485	-0.418483	0.774822
O	-1.139274	0.960389	-0.597385
H	0.136576	1.438731	-0.269523
N	1.167668	1.490122	0.165358
H	1.874344	0.805267	-0.134022
H	1.316039	1.891565	1.086576
H	1.573340	-1.249432	0.095280
O	2.421479	-0.966671	-0.267406
H	2.554911	-1.523256	-1.034931

DTS3 B3LYP/6-311+G(2df,2p)

O	-2.157370	-0.887651	-0.080906
N	-1.181514	-0.190973	0.039286
O	-0.169503	-0.528404	0.711848
O	-1.141685	0.977640	-0.541751

H	-0.006510	1.395890	-0.252201
N	1.145455	1.551883	0.171679
H	1.901281	0.938734	-0.148674
H	1.337910	1.953398	1.084884
H	1.665629	-1.267967	0.030176
O	2.527272	-0.932865	-0.254167
H	2.884382	-1.576187	-0.871142

DCR4 QCISD/6-311+G(2df,2p)

N	1.327338	-0.213523	0.008379
O	1.103943	1.118301	-0.056357
H	0.113477	1.246753	-0.014549
O	2.474679	-0.541582	-0.023319
O	0.351763	-0.936829	0.093183
N	-1.562117	1.657981	0.041483
H	-2.269705	0.918991	0.053406
H	-3.063101	-1.876993	0.217381
H	-2.054689	2.550152	0.060625
O	-2.606898	-1.095920	-0.092549
H	-1.670421	-1.301873	-0.033559

DCR4 QCISD/6-311+G(d,p)

O	-2.460941	-0.579735	0.030827
N	-1.320597	-0.219947	-0.010867
O	-0.324528	-0.918941	-0.108349
O	-1.134129	1.123644	0.054536
H	-0.147840	1.274508	0.006155

N	1.525083	1.648598	-0.046088
H	2.209672	0.881621	-0.045180
H	2.051048	2.526216	-0.057444
H	1.709802	-1.287295	0.216069
O	2.647296	-1.092821	0.132106
H	2.924335	-1.652789	-0.593871

DCR4 B3LYP/6-311+G(2df,2p)

O	-2.521846	-0.744571	-0.125239
N	-1.385822	-0.365558	-0.064484
O	-0.374674	-1.047822	-0.148906
O	-1.226452	0.974406	0.119156
H	-0.228656	1.174777	0.147508
N	1.360944	1.689419	0.211634
H	2.125083	1.010552	0.108436
H	1.771332	2.615471	0.341928
H	1.654823	-1.209716	-0.146683
O	2.578768	-0.935036	-0.082405
H	3.101573	-1.637094	-0.476045

DTS4 QCISD/6-311+G(2df,2p)

N	0.097215	0.032816	-0.082398
O	0.283432	0.327991	1.082629
O	0.914938	-0.148490	-0.932289
O	-1.191651	-0.122709	-0.492071
H	-1.755475	0.075897	0.283313

N	-3.889175	-1.128794	0.512806
H	-4.053911	-0.858654	1.485908
H	-4.617810	-1.818039	0.312038
O	-2.336206	1.034037	1.912107
H	-1.414249	1.095963	2.175243
H	-2.647059	1.938145	1.857256

DTS4 B3LYP/6-311+G(2df,2p)

N	0.099958	0.041142	-0.076675
O	0.281799	0.335770	1.093271
O	0.925114	-0.137595	-0.925302
O	-1.199459	-0.117933	-0.499456
H	-1.785321	0.065660	0.273011
N	-3.884772	-1.151569	0.530403
H	-4.103233	-0.859642	1.489028
H	-4.609865	-1.836839	0.293647
O	-2.332310	1.050718	1.925344
H	-1.393753	1.069314	2.152644
H	-2.608109	1.969141	1.858629

DCR5 B3LYP/6-311+G(2df,2p)

N	1.755231	0.181226	-0.394501
O	1.234733	1.261723	-0.611621
O	2.783815	-0.245191	-0.843683
O	1.100551	-0.663184	0.464079
H	0.260732	-0.177968	0.742991
N	-3.593512	-0.640097	0.604149

H	-3.902807	-1.099385	-0.257183
H	-4.341707	-0.805735	1.283297
O	-1.079475	0.618295	1.184168
H	-1.000455	1.435735	0.681918
H	-1.947051	0.225347	0.959299

References

(1) Anglada, J. M.; Olivella, S.; Solé, A. Unexpected Reactivity of Amidogen Radical in the Gas Phase Degradation of Nitric Acid. *J. Am. Chem. Soc.* **2014**, *136* (19), 6834–6837. https://doi.org/10.1021/ja501967x.
